# Carbon Emission Based Predictions of Anthropogenic Impacts on Groundwater Storage at Typical Basins in 2050

**DOI:** 10.34133/research.0680

**Published:** 2025-06-02

**Authors:** Ying Zhao, Jiabin Ma, Yuelei Li, Kui Cheng, Meiling Zhang, Zhuqing Liu, Fan Yang

**Affiliations:** ^1^ School of Water Conservancy & Civil Engineering, Northeast Agricultural University, Harbin 150030, China.; ^2^ International Cooperation Joint Laboratory of Health in Cold Region Black Soil Habitat of the Ministry of Education, Harbin 150030, China.; ^3^ School of Environment, Harbin Institute of Technology, Harbin 150090, China.; ^4^ College of Engineering, Northeast Agricultural University, Harbin 150030, China.

## Abstract

Understanding the impacts of anthropogenic activities on groundwater is crucial for its management and utilization. However, predicting anthropogenic impacts on groundwater remains challenging due to their complexity. As any anthropogenic activity generates carbon emissions, we employed carbon emissions to characterize the intensity of anthropogenic activities to predict groundwater storage variations. Carbon emission–groundwater machine learning models indicate that groundwater storage will increase in Rhine Valley (7.3% ± 1.9%), the Great Lakes Basin (6.7% ± 4.3%), and Pearl River catchments (1.8% ± 1.5%) in the next 3 decades, but it will continue to decline in Yangtze River catchments (−13.7% ± 3.4%), with *R*^2^ ranging from 0.916 to 0.995. Furthermore, the existing groundwater protection measures of Yangtze River catchments will not be sufficient to compensate for future declines in groundwater storage caused by anthropogenic activities (5.9% ± 4% decrease in 2050), indicating the necessity of more effective measures. This study developed a method to predict the impacts of anthropogenic activities on groundwater, thus overcoming an important obstacle in predicting groundwater behavior, which is crucial for the utilization and management of groundwater resources. The methodology developed in this study for predicting the impacts of anthropogenic activities on groundwater will raise awareness of the link between anthropogenic activities and groundwater and lead to in-depth research on anthropogenically driven groundwater prediction studies. This will overcome substantial barriers to predicting groundwater behavior, which is critical for groundwater resource use and management.

## Introduction

Groundwater is an important freshwater resource that provides 50% of the world’s drinking water [1], and it is essential for daily life, agricultural irrigation, and industrial processes [2]. Currently, approximately 40% of the world’s irrigation water comes from groundwater. The growing demand for food places substantial pressure on global groundwater resources [3,4]. Excessive anthropogenic extraction can cause various water security issues, such as groundwater resource shortages, ecological environment deterioration, and land subsidence [5–7]. It is crucial to determine the impacts of anthropogenic activities on groundwater and develop targeted strategies to maintain groundwater stability [4,8]. However, predicting anthropogenic effects on groundwater storage is challenging, due to the diversity of human activities and the lack of standardized quantification methods.

Most groundwater prediction studies rely on time-series data of groundwater alone or in combination with climatic factors, such as precipitation and temperature [9–12]. We collected 91 studies on groundwater prediction conducted from 2000 to 2023, all of which used natural factors as inputs (Fig. S1). While these studies can predict groundwater variations by considering changes in meteorological or hydrological factors, they ignore another key factor—anthropogenic activity [13,14]. Quantifying the contributions of anthropogenic factors is a complex task. Scientists have developed methods to quantify human activities using land-use, nighttime lighting, and population density datasets [15,16]. Numerical models such as Modflow, Community Water Model (CWatM), Improvement of the Slope Change Ratio of Cumulative Quantity (ISCRCQ), and Global Gradient-Based Groundwater Model (G^3^M) provide research methods for exploring the impacts of anthropogenic activities on groundwater [17–21]. Nevertheless, these methods cannot fully capture the impacts of various industries, making it challenging to predict groundwater variations influenced by anthropogenic factors.

Since the Industrial Revolution, fossil fuels have been extensively used in all aspects of human society. Anthropogenic activities inevitably produce carbon emissions [22,23]. Consequently, we build upon previous research and utilize the carbon emission index to quantify the impact of anthropogenic activities [24]. In this study, we analyzed and predicted the impact of anthropogenic activities on groundwater using data on carbon emissions and groundwater storage. To minimize the potential impacts of climate change, we selected 4 representative basins with a small range of latitudinal variation: Rhine Valley (RV), the Great Lakes Basin (GLB), Pearl River catchments (PRC), and Yangtze River catchments (YZRC) (Fig. S2). Groundwater and carbon emission time-series data were collected from 2003 to 2018, resulting in a total of 1,392, 4,240, 2,672, and 10,576 samples for each basin, respectively. Subsequently, we use 4 classical machine learning models, namely, convolutional neural networks (CNNs), random forests (RFs), extreme gradient boosting (XGBoost), and support vector regression (SVR), to characterize their relationship [13]. In the training and testing processes, carbon emissions resulting from anthropogenic activities were used as input variables, whereas groundwater storage was used as an output variable. The models that best represented these relationships were selected for further prediction tasks. Based on these findings, we designed 3 fundamental pathways (including 7 carbon emission scenarios) to predict groundwater storage. Sensitivity analysis was employed to quantify the sensitivity response of groundwater storage to carbon emissions in different sectors. Uncertainty analysis and similar region validation were used to analyze the predictive performance of the model. In addition, groundwater protection measures are evaluated for basins where groundwater storage declined during the predicted period. This study enables the prediction of groundwater storage influenced by anthropogenic factors and provides a basis for groundwater planning and utilization.

## Results

### Impacts of anthropogenic factors on groundwater storage

**Fig. 1.**
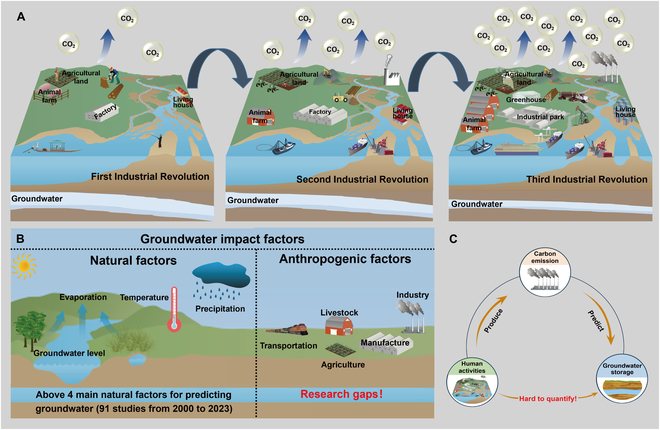
Predicting anthropogenic impacts on groundwater based on carbon emission data. (A) The growing impacts of anthropogenic activities on groundwater storage and carbon emissions since the first Industrial Revolution. (B) In past groundwater prediction studies, researchers have focused on the influence of natural factors and ignored the role of anthropogenic factors. (C) Carbon emissions are a useful measure of anthropogenic factors for groundwater prediction.

To address the complexity of anthropogenic activities, carbon emissions are proposed to quantify the intensities of various anthropogenic activities [25]. Since the beginning of the Industrial Revolution, the intensity of anthropogenic activity has increased, resulting in a corresponding increase in carbon emissions and groundwater consumption (Fig. 1A). However, previous research studies were not sufficiently accurate or they deviated from the actual situation because they ignored the impact of anthropogenic activity on groundwater (Fig. 1B). Anthropogenic activities are essential factors for groundwater storage variation, and they need to be considered in groundwater analysis. Thus, this study uses carbon emissions to predict groundwater storage (Fig. 1C). In the following sections, carbon emissions are adopted as a bridge to explore the relationship between anthropogenic factors and groundwater storage variations.

### Groundwater storage predictions by carbon emission

Carbon emission data (14 categories) and groundwater storage data from 2003 to 2018 for 18,880 samples were collected to analyze their relationship (carbon emissions and groundwater spatial and temporal characteristics are detailed in Note S1 and Figs. S2 to S9). The spatial distribution of groundwater storage demonstrates significant regional variations in the research basins, reflecting the combined influences of natural conditions and anthropogenic activities. The RV basin displays a substantial storage capacity relative to its central urban areas, resulting from (a) recharge from southern mountainous catchments and (b) high-permeability Quaternary alluvial aquifers in northern regions. GLB maintains enhanced storage potential in its northeastern sector due to optimal infiltration conditions sustained by a forest ecosystem. The PRC basin shows a reduced storage capacity in southern urbanized areas, where impervious surface coverage significantly restricts infiltration processes. The YZRC basin demonstrates minimal storage retention in its northwestern topographic transition zone, where steep gradients promote rapid groundwater discharge.

In terms of the time-series change in carbon emissions from 2003 to 2018, RV and GLB changed less and remained flat overall, while carbon emissions from PRC and YZRC had an upward trend. In addition, the largest source of carbon emissions for RV, PRC, and YZRC is “energy industry (ENE)”, while that for GLB is “road transport (TRO_noRES)”. The spatial distribution of carbon emissions is higher in the center and north of RV; higher in the south of GLB; higher in the southeast of PRC, but not significantly so; and higher in the east of YZRC and lower in the west. It is noteworthy that the agricultural carbon emissions of RV, PRC, and YZRC are more evenly distributed across the basin.

#### Relationships between carbon emissions and groundwater storage by machine learning model simulations

**Fig. 2.**
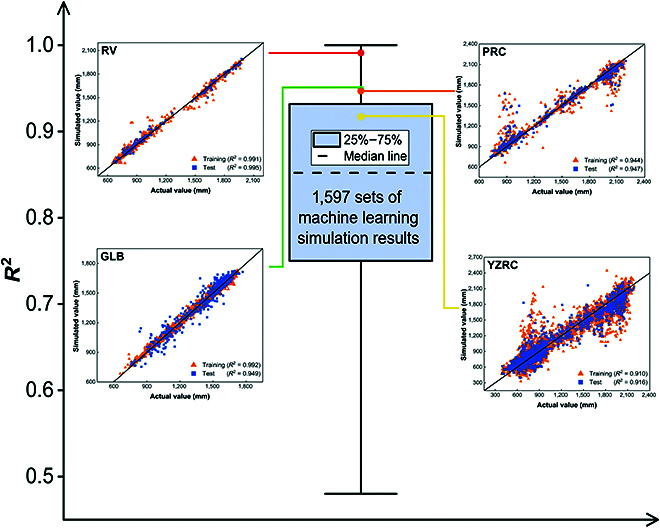
High accuracy of the constructed machine learning model. Comparison of machine learning accuracy in this research with previous 1,597 sets of groundwater predictions (2000 to 2023). Training and test accuracy of machine learning in Rhine Valley (RV), Great Lakes Basin (GLB), Pearl River catchments (PRC), and Yangtze River catchments (YZRC).

**Fig. 3.**
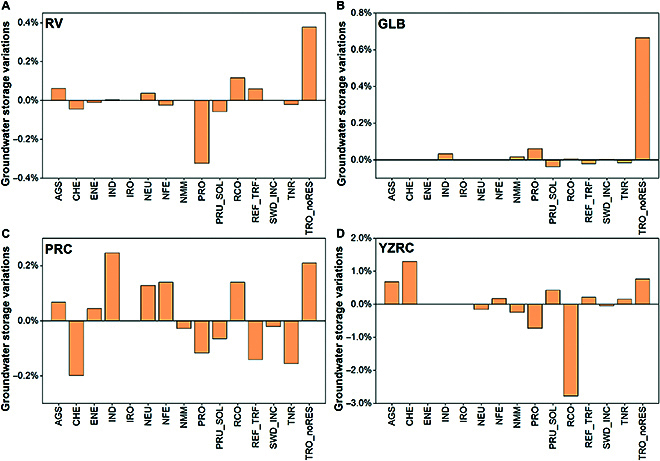
Sensitivity response of groundwater storage in different basins. (A) RV. (B) GLB. (C) PRC. (D) YZRC. Impact of a 5% decline in carbon emissions on groundwater storage in different categories. AGS, agricultural soils; CHE, agricultural waste burning; ENE, energy industry; IND, combustion in the manufacturing industry; IRO, iron and steel production; NEU, non-energy use of fuels; NFE, production of nonferrous metals; NMM, production of nonmetallic minerals; PRO, fuel production/transmission; PRU_SOL, production and use of other products; RCO, residential; REF_TRF, oil refineries; SWD_INC, solid waste disposal; TNR, non-road transport; TRO_noRES, road transport.

The SVR model exhibited the best performance for RV, PRC, and YZRC, and the XGBoost model exhibited the best performance for GLB, with *R*^2^ values of 0.995, 0.947, 0.916, and 0.949, respectively. This study comparatively analyzed 91 groundwater prediction studies with 1,597 datasets, and the *R*^2^ of the constructed model exceeded that of the majority of the previous studies (Fig. 2). Only the model with the best performance is used for prediction in the following section. The training and test results for all the machine learning models are provided in the Supplementary Materials (Table 1 and Figs. S10 to S13). The modeling results show that most of the groundwater storage prediction data have relative errors of less than 20%, and the results for RV and PRC are even less than 10% (Fig. S14). Furthermore, the discrepancies in the predicted groundwater storage are considerable for a few of the samples. The limited amount of data may be a contributing factor, as the model is unable to resolve the correlation between groundwater and carbon emissions in regions exhibiting “extreme” values. For groundwater predictions, most groundwater storage predictions are sufficiently accurate to be subjected to in-depth analysis. The results of the sensitivity analysis indicate that the sectors of agricultural soils (AGS) and road transport (TRO_noRES) can exert a considerable negative influence on groundwater storage. In general, the impact of carbon emissions on groundwater storage varies across different regions. The impact on YZRC is more pronounced in comparison to those on the other 3 basins (Fig. 3). This indicates that YZRC is utilizing a larger proportion of groundwater for human activities and the pressure on groundwater sustainability is more pronounced.

**Table 1.** Machine learning model outputs. Boldface highlights the accuracy of the fit of the optimal model for each basin.

Basins	Dataset	*R* ^2^				RMSE			
		CNN	RF	XGBoost	SVR	CNN	RF	XGBoost	SVR
RV	Training	0.961	0.988	0.994	**0.991**	0.062	0.033	0.024	**0.029**
	Test	0.923	0.952	0.964	**0.995**	0.079	0.067	0.057	**0.021**
GLB	Training	0.800	0.929	**0.992**	0.820	0.106	0.064	0.022	**0.100**
	Test	0.771	0.881	**0.949**	0.801	0.114	0.080	0.053	**0.107**
PRC	Training	0.927	0.904	0.996	**0.944**	0.088	0.100	**0.020**	0.077
	Test	0.855	0.801	0.816	**0.947**	0.123	0.148	**0.138**	0.075
YZRC	Training	0.804	0.739	0.979	**0.910**	0.128	0.148	0.042	**0.087**
	Test	0.699	0.665	0.763	**0.916**	0.162	0.167	0.140	**0.082**

CNN, convolutional neural network; RF, random forest; XGBoost, extreme gradient boosting; SVR, support vector regression; RMSE, root mean square error

#### Scenarios of prediction

To simulate the intensity of human activities under possible future conditions, 3 pathways (carbon neutrality and global warming of 1.5 and 2 °C) including 7 potential carbon emission scenarios are designed in Table 2. The carbon-neutral scenario (CNS), a potential carbon emission scenario with the basic purpose of carbon neutrality, was designed based on government documents and relevant research information from the region or country where each basin is located. In addition, this study uses 6 potential carbon emission scenarios designed by the Intergovernmental Panel on Climate Change (IPCC) to achieve the goal of controlling global temperature rise at 1.5 °C and below 2 °C above pre-industrial levels by the end of this century [26]. The 6 scenarios are Below-1.5 °C (IPCC1.5B), 1.5 °C-low-OS (IPCC1.5L), 1.5 °C with no or limited OS (IPCC1.5W), 1.5 °C-high-OS (IPCC1.5H), Lower-2 °C (IPCC2L), and Higher-2 °C (IPCC2H) (Table S1).

**Table 2.** Potential carbon emission scenarios

Scenario	Pathway characteristic
**CNS**
Carbon-neutral scenario	This scenario is designed according to the carbon emissions planned for each period in the area where each basin is located to achieve carbon neutrality.
**IPCC1.5B, IPCC1.5L, IPCC1.5W, and IPCC1.5H**	
Below-1.5 °C	This scenario has a high probability of keeping global warming below 1.5 °C by the end of the 21st century.
1.5 °C-low-OS	This scenario is likely to keep global warming below 1.5 °C by the end of the 21st century, but the odds are that it will exceed that limit by about 0.1 °C before then.
1.5 °C with no or limited OS	Carbon emissions in this scenario fall by about 45% in 2030 compared to those in 2010 and are net zero by 2050.
1.5 °C-high-OS	This scenario is likely to keep global warming below 1.5 °C by the end of the 21st century, but the odds are that it will exceed that limit by about 0.1–0.4 °C before then. Moreover, this scenario is more likely to exceed 1.5 °C compared to 1.5 °C-low-OS.
**IPCC2L and IPCC2H**	
Lower-2 °C	This scenario has a high probability of keeping global warming below 2 °C.
Higher-2 °C	This scenario has a low probability of keeping global warming below 2 °C (less likely than Lower-2 °C, but still over 50%).

IPCC1.5B, IPCC1.5L, IPCC1.5W, IPCC1.5H, IPCC2L, and IPCC2H are the potential carbon emission scenarios predicted by the Intergovernmental Panel on Climate Change (IPCC; see Note S2 for details) [43]. OS: Temporarily exceeding pre-industrial levels until some point before 2100, after which it returns to values below 1.5 °C above that level—known as overshoot. Therefore, the carbon emission scenarios assumed in these scenarios may differ somewhat from the actual situation in each basin, which is an “idealized” scenario.

#### Similar basin validation

**Fig. 4.**
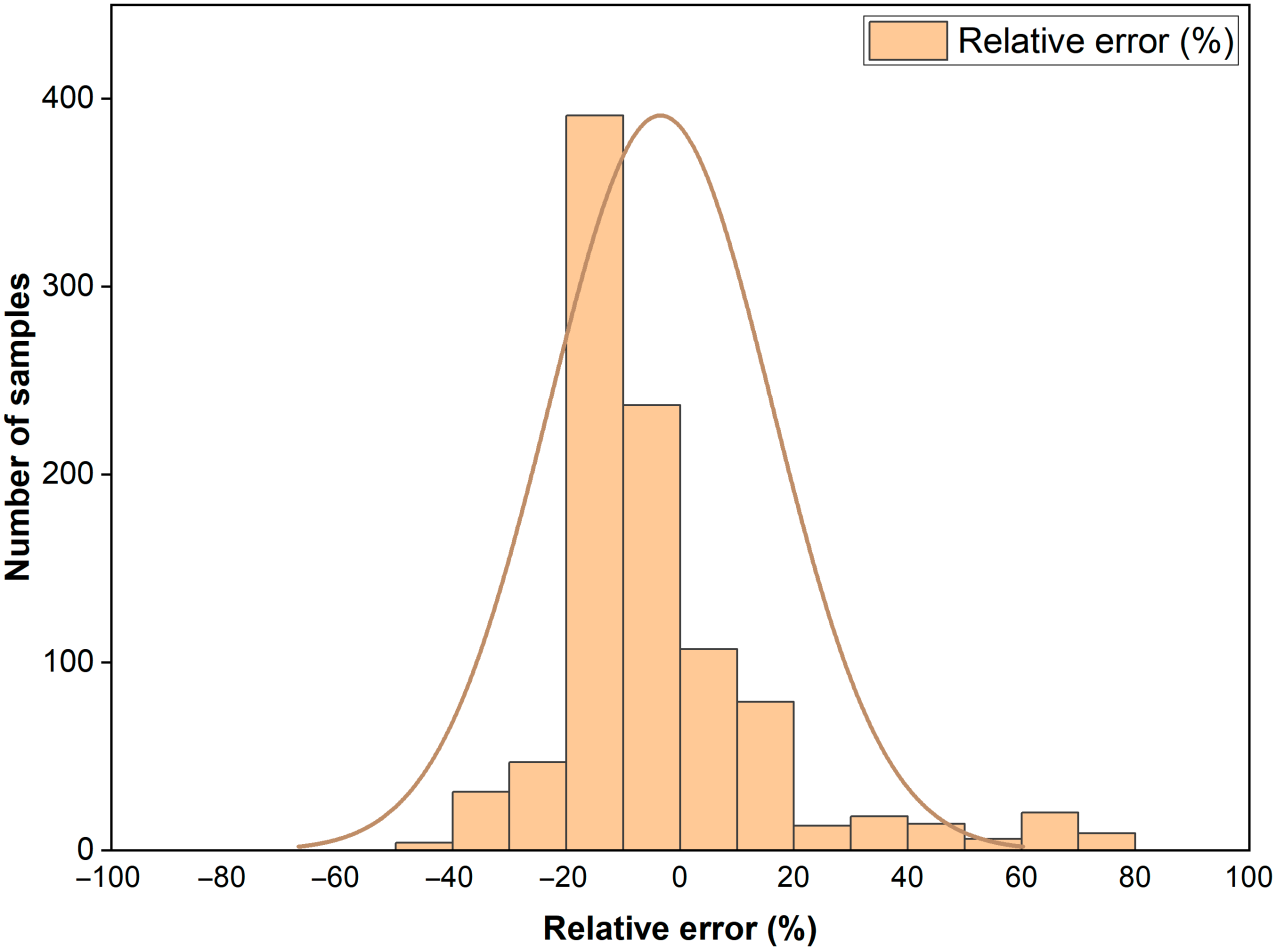
Relative error between predicted and true values in the validation area.

To verify the predictive performance of the model under the scenarios of future changes, the Southeast River Basin in China, which has natural conditions similar to those of PRC, was selected as a validation area. The mean relative error of the predicted groundwater storage within the validation area was 14.7% in absolute terms. Notably, the relative errors associated with the predicted groundwater storage values were predominantly within the range of ±20% across the majority of the sample points (Fig. 4). This indicates the effectiveness of the proposed model in predicting groundwater storage under potential scenarios.

#### Uncertainty analysis

**Fig. 5.**
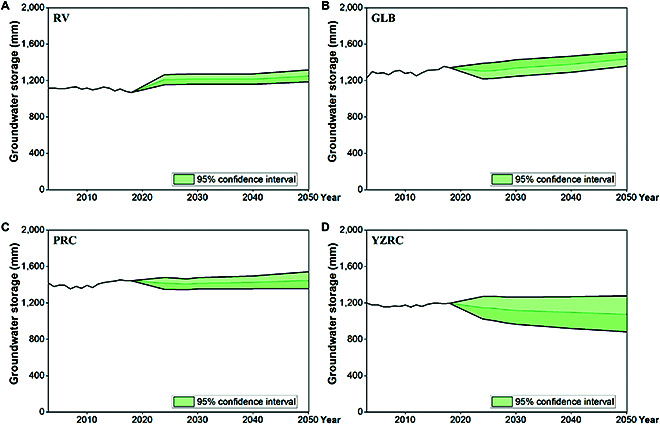
Uncertainty analysis results of all basins in CNS. (A) Uncertainty analysis results of RV. (B) Uncertainty analysis results of GLB. (C) Uncertainty analysis results of PRC. (D) Uncertainty analysis results of YZRC.

The results of the uncertainty analysis demonstrated that the uncertainty ranges of RV, GLB, and PRC were ±5.1%, ±6.6%, and ±8.1%, respectively, within a 95% confidence interval (Fig. 5A to C). The uncertainty range for YZRC is higher compared to those for the other 3 basins at ±16.7% (Fig. 5D). The results of the uncertainty analysis for all scenarios are shown in Figs. S16 to S19. This finding indicates that machine learning models for individual basins have the capacity to accurately predict groundwater storage under future changes in energy and water use.

#### Temporal and spatial distribution of groundwater storage over the prediction period

**Fig. 6.**
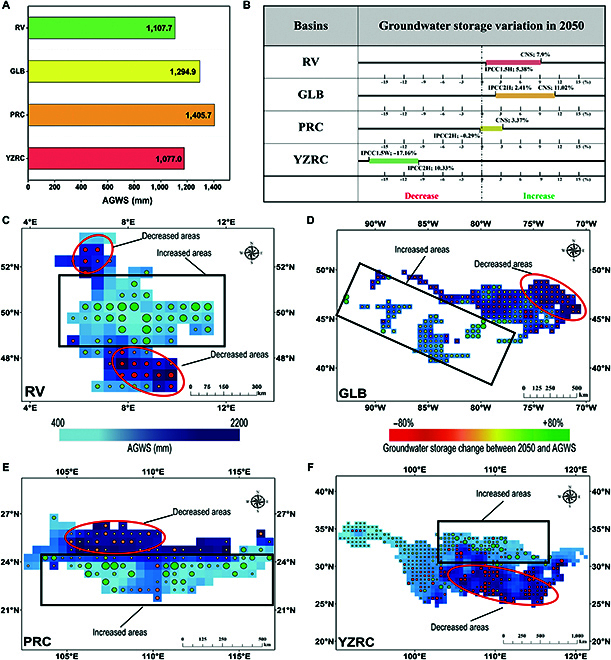
Spatial and temporal groundwater storage variation over the prediction period. (A) Average groundwater storage from 2003 to 2018 (AGWS). (B) Temporal groundwater storage variation in 2050 of the 7 scenarios. (C) RV. (D) GLB. (E) PRC. (F) YZRC. Change percentage in groundwater storage for each grid sample in 2050 relative to AGWS. The size of the point indicates the magnitude of groundwater storage variation; the larger the point, the greater the variation.

Predictions of groundwater storage variations in 2050 under 7 different scenarios based on carbon emission data showed that the anthropogenic intensity in CNS is more conducive to groundwater storage (Figs. S15 to S18). There were significant increases in groundwater storage in RV, GLB, and PRC (9.26%, 11.02%, and 3.37%, respectively) and a decrease in YZRC of only 10.49%, compared to the average groundwater storage from 2003 to 2018 (hereafter AGWS; the AGWS of all basins is shown in Fig. 6A). Furthermore, the scenarios with the most unfavorable groundwater storage variations in RV, GLB, PRC, and YZRC groundwater are IPCC2L (5.38%), IPCC2H (2.41%), IPCC2H (−0.29%), and IPCC1.5W (−17.16%), respectively (Fig. 6B).

By comparing the spatial distributions of the groundwater storage variations in the 4 basins across all 7 scenarios, we found that the spatial distributions were generally consistent (Figs. S19 to S24). The differences lie only in the amount of groundwater storage variation and extent of the growth areas. Therefore, we selected CNS as a representative scenario for the following analysis. The RV heartland experienced significant growth (groundwater storage increased by approximately 40% to 80% of AGWS), while there was a slight decline in the northern and southern regions (approximately 20% to 40%). Groundwater storage in GLB showed an overall increasing trend, with a higher rate of increase near the center of the lake (more than 40%), but a tiny decreasing rate in the northeastern region (approximately 0% to 20%). The groundwater storage area of PRC can be divided by an east–west central line, with an ascending zone in the south (approximately 60% to 100%) and a descending zone in the north (approximately 0% to 40%). Groundwater in the northern part of YZRC showed a higher increase (more than 80%), and in the northwestern part of YZRC, there was a moderate increase (approximately 0% to 20%). However, groundwater storage in most areas of YZRC showed a downward trend, especially in the central region (approximately 80% to 100%) (Fig. 6C to F).

### Analysis of spatial and temporal variations in groundwater storage

During the predicted period, groundwater storage in most basins exhibited an increasing trend, except for YZRC (Table S2). Specifically, RV exhibited a favorable upward trend in groundwater storage over the next 3 decades in all scenarios. Groundwater storage in GLB also displayed an increasing trend in most scenarios but remained lower than that of AGWS at all predicted time points in IPCC2H. Groundwater storage in PRC will decline until 2030, after which it will increase in all scenarios. This is consistent with the peak time of carbon emissions in China, and the intensity of abatement reform will change dramatically around 2030 [27,28]. Additionally, significant increases in groundwater storage were observed along the southern coast of PRC, which may be the result of seawater recharge [29,30]. Previous studies have shown significant seawater intrusion in southern PRC [31], and the predicted results surmise that the seawater intrusion phenomenon has become more serious. In contrast, YZRC exhibits a consistent downward trend that becomes more significant after 2030. The reduction in groundwater storage is particularly noticeable in the central and southern regions of YZRC, which are characterized by high forest cover. Most grids with declining groundwater storage were located in forested areas, and grids with declines of more than 20% were located entirely within forested areas (Fig. S25). Studies have also shown that forest carbon sinks consume large amounts of groundwater and that forests have been the dominant source of carbon sinks in China over the past few decades [32,33]. This finding suggests that groundwater storage should be considered when reducing carbon emissions from forest carbon sinks.

In terms of spatial regulation, all basins exhibited a common feature that grid samples with high original groundwater storage generally presented a decreasing trend (south and north of RV, northeast of GLB, and south of PRC and YZRC). In particular, groundwater decline is generally concentrated in the Netherlands, eastern Switzerland, southern Germany, Austria, and Liechtenstein in RV; the border between Ontario and southern Quebec in Canada in GLB; and in Jiangxi, Hunan, Guizhou, and north-central Sichuan in PRC and YZRC (Fig. S26). Furthermore, the differences in the total groundwater storage variations among the 7 scenarios were mainly due to significant localized changes. This was particularly evident in YZRC, where an unusual decline in groundwater storage in the southern part has resulted in a decreasing trend in total groundwater storage. Therefore, all scenarios must consider the localized impacts of groundwater storage decline, particularly in areas with high original groundwater storage.

### Assessment of groundwater protection measures in YZRC

**Fig. 7.**
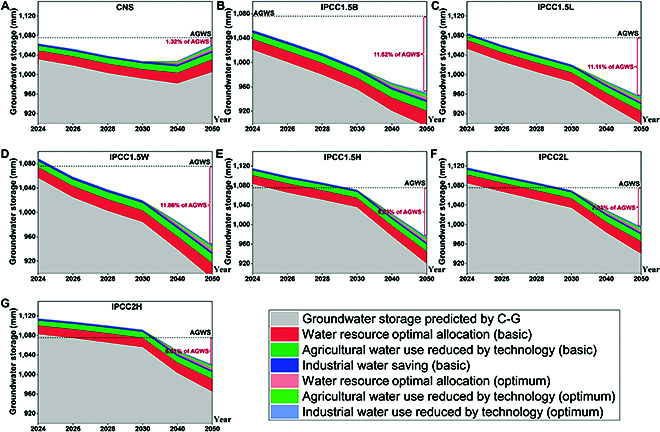
Groundwater storage by current water protection measures in YZRC. The gray area indicates the groundwater storage predicted by carbon emission. The amount of groundwater storage in YZRC under all predicted scenarios. (A to G) Seven carbon emission scenarios. C-G stands for the carbon emission–groundwater prediction model.

The results of this study indicate that groundwater storage is lower than the AGWS at the majority of the predicted time points in YZRC, which serves to highlight the urgency of the problem of groundwater overexploitation in YZRC. In fact, the Chinese government has long been concerned about and has taken engineering measures dedicated to the sustainable development of water resources in YZRC, such as the Yangtze-Han River Diversion Project and the Three Gorges Water Conservancy Project. Consequently, an additional assessment was conducted to determine the potential of existing water regulation or water conservation measures to address the human-induced decline in groundwater storage in YZRC. This section further assesses the potential for groundwater resource recovery in YZRC through 3 key areas, water resource regulation, agricultural water saving, and industrial water saving, under 2 scenarios, basic and optimum. The results of the assessments indicate that the groundwater protection measures implemented by YZRC are ineffective in restoring groundwater storage to its current levels in all scenarios (Fig. 7). The groundwater recovery in CNS is the most successful, although it remains below the AGWS of 1.32%. In contrast, the groundwater in IPCC 1.5W has the least bad recovery, below the AGWS of 11.86%. Despite the favorable outcomes of the groundwater protection measures implemented by YZRC, these measures are unable to offset the decline in groundwater levels caused by anthropogenic activities. YZRC still needs to take more effective measures to mitigate the potential groundwater crisis; the most important is to improve the efficiency of water use in agriculture. YZRC’s agriculture is widely distributed; the use of groundwater accounts for a large proportion of the total, but the efficiency of water use is relatively low. The issue of water use in agriculture is the key to the sustainability of groundwater in YZRC. It is notable that the groundwater storage of YZRC in CNS demonstrates a pronounced upward trend after 2040. This indicates that groundwater storage may return to its current levels at some point after the prediction period.

## Discussion

This study predicted the impact of anthropogenic activities on groundwater storage based on the carbon emission index. To minimize the potential impacts of climate change, 4 representative basins, with a small range of latitude variation, were selected as the study areas. The carbon emission–groundwater storage machine learning model predicted a continuously increasing trend in groundwater storage in RV, GLB, and PRC under 7 potential carbon emission scenarios and a continuously decreasing trend in groundwater storage in YZRC due to differences in the intensity of anthropogenic activities (*R*^2^ ranging from 0.916 to 0.995). Among all scenarios, CNS is the most favorable for groundwater sustainability in typical basins. Additionally, an evaluation was conducted to assess the potential of the groundwater protection strategy to restore groundwater storage in YZRC. The results demonstrate that the current groundwater protection measures are still unable to halt the regional groundwater depletion trend. Consequently, YZRC requires the implementation of more effective water conservation measures. As most categories of carbon emissions impact groundwater storage, it is recommended that the carbon emission proportion be optimized to realize groundwater storage recovery and carbon-neutral goals.

This research used carbon emission data to quantify anthropogenic impacts on groundwater storage and explored trends in basin-scale groundwater variations under different scenarios. The results contribute to a deeper understanding about changes in groundwater response under the impacts of anthropogenic activities. These will enable stakeholders to develop targeted groundwater management strategies that balance human development needs with groundwater sustainability. In the future, research on the impact of using green energy and changes in infrastructure on groundwater variations is recommended.

## Methods

### Data

This study used groundwater storage data (0.25° × 0.25°) from the GLDAS_CLSM025_DA1_D dataset (https://disc.gsfc.nasa.gov) [34,35]. Carbon emission data (0.1° × 0.1°) were obtained according to the EDGAR v6.0 Greenhouse Gas Emissions dataset (https://data.jrc.ec.europa.eu) [36]. In addition, the groundwater storage data (0.25° × 0.25°) and carbon emission data (0.1° × 0.1°) were downscaled to 0.5° × 0.5° on the same scale using the ArcMap 10.2 aggregation toolbox (Fig. S27). There are 87, 265, 167, and 661 grid samples (0.5° × 0.5°) in RV, GLB, PRC, and YZRC, respectively, in the combined 0.5° × 0.5° dataset. Moreover, the groundwater storage data were changed from daily to annual to align the time scales of groundwater storage data and carbon emission data. The unit of groundwater storage obtained from the GLDAS dataset is “mm” (equivalent water thickness), and it is used in the subsequent analysis discussion. Following, the data were screened for extreme values using the interquartile range outlier processing technique, and the data were processed using min–max normalization. Finally, the data were shuffled and 80% of the data were divided into a training set and fed into the model for training, while the remaining 20% were used as a test set to validate the accuracy of the model.

### Convolutional neural networks

CNNs are commonly used in image recognition and classification tasks [37,38]. This model is widely used in natural language processing, recommendation systems, and speech recognition. It is also helpful for predicting groundwater storage. The typical CNN model consists of an input layer, a convolution layer, a pooling layer, a full connection layer, and an output layer. The input layer can receive input in vector and matrix forms, allowing it to process various types of data without breaking the internal structure. In the convolutional layer, the convolutional check traverses the input data with the same weight for each local region, so that this layer is only partially connected to the neurons of the previous layer. Using average or maximum pooling, the pooling layer reduces the feature size by nonlinear down sampling. The pooling mechanism can significantly reduce network scale and improve computing efficiency. The neurons of the fully connected layer connect all the elements of the upper layer and convert the features into one-dimensional vectors for easy processing. The output layer (usually a fully connected layer) generates the final output of the model. In practice, CNNs also contain dropout and activation functions. The dropout function prevents overfitting by temporarily removing some network units with a certain probability during training. The activation function is mainly used to fit the nonlinear mapping of the features extracted by the convolutional layer, to improve the ability of CNNs in nonlinear data processing. For the CNNs, we equally use MATLAB R2022a.

### Random forest

RF was proposed by Breiman [39], which is a specific implementation of the bagging method. It trains multiple decision trees and then merges the results to produce the final result. RFs can be used for classification as well as regression. They mainly consist of the choice of decision tree type, where decision trees of a specific category are selected based on a specific task. For regression, the RF prediction is the average of the outputs of all decision trees. RF models deal with random binary trees that use a subset of the observed data via a bootstrapping technique, where a random subset of the training dataset is sampled from the original dataset and used to develop the model. In addition, the introduction of randomness makes RF less prone to overfitting and also gives RF excellent anti-noise capabilities. The RF model was implemented using SPSSPRO, an online data analysis platform.

### Extreme gradient boosting

XGBoost is a distributed gradient boosting toolkit, and it uses the gradient boosting framework to construct machine learning algorithms [40]. It is an efficient implementation of the gradient boosting decision tree (GBDT). Unlike GBDT, XGBoost adds a regularization term to the loss function. Moreover, because some loss functions are difficult-to-calculate derivatives, XGBoost uses the second-order Taylor expansion of the loss function as the fitting of the loss function. The algorithm is derived from the idea of “boost”, which combines all predictions of a set of “weak” learners to develop “strong” learners through an additive training strategy. The purpose of the XGBoost model is to prevent overfitting while minimizing computational costs. XGBoost offers parallel tree boosting (also known as GBDT or gradient boosting machine) to address a wide range of data science issues quickly and accurately. This study employs gbtree with a learning rate of 0.05 and controls the maximum depth of the tree and sampling conditions to adjust the model. Then, we add the L2 regular term to prevent the model from overfitting. As with RF, XGBoost is implemented via SPSSPRO.

### Support vector regression

SVR is an important branch of the support vector machine, which is a supervised learning model for regression analysis [41,42]. Although less popular than the support vector machine, SVR has proven to be an effective tool for real-valued function estimation. The computational complexity of SVR does not depend on the dimension of the input space. Moreover, it has excellent generalization capability with a high prediction accuracy. The SVR model in this study used the radial basis function, and the gamma function is 1/*k*. The loss function “*p*” is chosen to be 0.01 to prevent overfitting. Meanwhile, we adjust the penalty factor and radial basis function to make the model reach the optimal condition. To build and apply SVR models, we use MATLAB R2020a and its Libsvm Toolbox.

### Genesis of the scenario

More detailed design ideas for scenarios can be found in Note S2.

### Groundwater protection assessment

More detailed design ideas for scenarios can be found in Note S3.

### Uncertainty analysis

Uncertainty analysis is defined as a systematic process of assessing model uncertainty. Bootstrap is a statistical resampling technique used to estimate the distributional characteristics of a sample statistic. It estimates the statistical properties of an aggregate by sampling multiple subsamples from the original sample in a relaxed manner and computing the statistic for each subsample. The number of resamples employed in this study is 100. The uncertainty analysis is implemented through MATLAB R2022a.

## Data Availability

The download links for the 0.5° × 0.5° carbon emission and groundwater storage time-series data have been uploaded to GitHub (https://github.com/MAJIABINNN/Predictions-of-anthropogenic-impacts-on-groundwater-storage-at-typical-basins). The other original data can be obtained from the authors upon reasonable request.

## Supplementary Materials

Supplementary 1 Notes S1 to S4 Figs. S1 to S27 Tables S1 to S3
