# Supplementary material for: Carbon Emission Based Predictions of Anthropogenic Impacts on Groundwater Storage at Typical Basins in 2050
Source: Research (Wash D C). 2025 Jun 2;8:0680. doi: 10.34133/research.0680 (PMC12129122; doi:10.34133/research.0680)
Supplement: Supplementary 1 — Notes S1 to S4 Figs. S1 to S27 Tables S1 to S3 [file research.0680.f1.pdf]

Carbon emission-based predictions of anthropogenic impacts on groundwater storage at typical basins in 2050

(Supporting information)

Ying Zhao[1,2†], Jiabin Ma[1,2†], Yuelei Li[3†], Kui Cheng[2,4], Meiling Zhang[1,2], Zhuqing Liu[1,2]*, Fan Yang[1,2]*

1. School of Water Conservancy & Civil Engineering, Northeast Agricultural University, Harbin 150030, China

2. International Cooperation Joint Laboratory of Health in Cold Region Black Soil Habitat of the Ministry of Education, Harbin 150030, China

3. School of Environment, Harbin Institute of Technology, Harbin, 150090, China

4. College of Engineering, Northeast Agricultural University, Harbin 150030, China

* Email: yangfan_neau@163.com and lzq@cau.edu.cn

† These authors contributed equally to this work.

TABLE of CONTENTS

Supplementary Note 1: Carbon emissions and groundwater storage data characterization 4
Supplementary Note 2: Genesis of the scenario 5
Supplementary Note 3: Protection of water resources 7
Water resources allocation 7
Agricultural water use reduced by technology 7
Industrial water use reduced by technology 8
Supplementary Note 4: Spatial matching of groundwater extraction and anthropogenic activities on the study unit 10
Supplementary Table: 11
Table S1 | Setting of carbon emission scenarios 11
Table S2 | Variation in all basins relative to AGWS at predicted time points 12
Table S3 | Distance of centralized groundwater sources in the Yangtze and Pearl River basins from the urban areas of the cities they supply 13
Supplementary Figure: 15
Supplementary Figure 1 | Utilization of various natural factors in groundwater prediction studies from 2000 to 2023. 15
Supplementary Figure 2 | Spatial characteristics of groundwater storage for all basins. 16
Supplementary Figure 3 | Carbon emissions variation in four basins. 17
Supplementary Figure 4 | Spatial distribution of carbon emissions from 14 categories and total carbon emissions in RV. 18
Supplementary Figure 5 | Spatial distribution of carbon emissions from 14 categories and total carbon emissions in GLB. 19
Supplementary Figure 6 | Spatial distribution of carbon emissions from 14 categories and total carbon emissions in PRC. 20
Supplementary Figure 7 | Spatial distribution of carbon emissions from 14 categories and total carbon emissions in YZRC. 21
Supplementary Figure 8 | Proportion of carbon emissions by category in four basins. ..22
Supplementary Figure 9 | Groundwater storage variations from 2003 to 2018. 23
Supplementary Figure 10 | CNN model output for all basins test sets. 24
Supplementary Figure 11 | RF model output for all basins test sets. 25
Supplementary Figure 12 | XGBoost model output for all basins test sets. 26
Supplementary Figure 13 | SVR model output for all basins test sets. 27
Supplementary Figure 14 | Relative errors in groundwater prediction models. 28
Supplementary Figure 15 | Predicted values of RV and results of uncertainty analysis. 29
Supplementary Figure 16 | Predicted values of GLB and results of uncertainty analysis.30
Supplementary Figure 17 | Predicted values of PRC and results of uncertainty analysis.31
Supplementary Figure 18 | Predicted values of YZRC and results of uncertainty analysis. 32
Supplementary Figure 19 | Groundwater storage variation in the IPCC1.5B. 33
Supplementary Figure 20 | Groundwater storage variation in the IPCC1.5L. 34
Supplementary Figure 21 | Groundwater storage variation in the IPCC1.5W. 35
Supplementary Figure 22 | Groundwater storage variation in the IPCC1.5H. 36
Supplementary Figure 23 | Groundwater storage variation in the IPCC2L. 37

Supplementary Figure 24 | Groundwater storage variation in the IPCC2H. 38
Supplementary Figure 25 | Forest cover and areas of declining groundwater storage. ...39
Supplementary Figure 26 | Basin Maps. 40
Supplementary Figure 27 | Grid sample data down-scaling diagram. 41
Supplementary References: 42

Supplementary Note 1: Carbon emissions and groundwater storage data characterization

The main sources of carbon emissions vary somewhat between basins, with Energy industry (ENE) being the largest source for RV and PRC, while GLB has the highest emissions in Road transport (TRO_noRES), and ENE and combustion in Manufacturing industry (IND) in YZRC each accounting for about one-third of emissions (Fig. S6a). Groundwater storage in the RV and GLB has increased since 2012, PRC has remained relatively flat, and YZRC has shown an overall fluctuating decline (Fig. S6b). Spatially, groundwater storage is more abundant in the central RV, northeastern GLB, northern PRC and south-central YZRC areas (Fig. S6c-f). The full names of the carbon emission categories can be found in the notes of Table S2-S4.

Supplementary Note 2: Genesis of the scenario

The region where the RV is located contains numerous European countries. Therefore, we select EU-related data for RV multi-scenario predictions. In addition, the GLB includes the U.S. and Canada, but the U.S. contributes additional carbon emissions to the GLB. Therefore, we refer to information on multi-scenario projections of the US. Both the PRC and the YZRC are basins of China. We design the scenario with respect to the literature and information on Chinese carbon emissions and make the same settings for both basins.

In designing potential carbon emission scenarios, we considered carbon neutrality and climate warming, two tasks and goals that are particularly urgent for the planet. Seven scenarios were designed on this basis: CNS, IPCC1.5B, IPCC1.5L, IPCC1.5W, IPCC1.5H, IPCC2L, and IPCC2H (Table S1). The CNS scenario is based on the carbon-neutral pathways designed by each basin's regional organizations or countries[1-3]. The other six scenarios are based on the data from the IPCC report "Global warming of 1.5 °C"[4].

The formula for determining carbon emissions at each time point during the projection period is as follows:

$$C_i = \frac{C_{PR} C_B}{C_{BR}} \quad (1)$$

Where $\boldsymbol{C_i}$ indicates carbon emissions at a forecast time point; $\boldsymbol{C_{PR}}$ indicates projected carbon emissions based on literature; $\boldsymbol{C_B}$ indicates carbon emissions in the basin in the base year; $\boldsymbol{C_{BR}}$ indicates carbon emissions in the base year in the reference.

In the choice of the time point of the scenario, we consider that China's carbon emissions have not yet peaked. The studies have suggested that China's carbon emissions will peak between 2028 and 2030[1]. Therefore, we design a time point every two years during 2024-2030. After that, according to various studies, the phase-out of carbon cuts would normally take place in 10-year increments. Therefore, we set a time point every ten years in the period 2030-2050.

Supplementary Note 3: Protection of water resources

The relevant groundwater data used in this section are from:

1) China Water Resources Yearbook (https://slnj.digiwater.cn/)
2) China Water Resources Bulletin (http://www.mwr.gov.cn/sj/tjgb/szygb/)
3) China Statistical Yearbook (http://www.stats.gov.cn/sj/ndsj/)
4) Yangtze River Basin and Southwest Rivers Water Resources Bulletin (http://www.cjw.gov.cn/zwzc/bmgb/szygb/)

Water resources allocation

Many water diversion projects are planned or have been built in YZRC to realize the problem of uneven distribution of water resources in YZRC and to reduce the excessive consumption of local groundwater resources. The YZRC has completed the Three Gorges Water Conservancy Hub Project, the Poyang Lake Water Conservancy Hub Project, the Middle and Lower Yangtze River Irrigation District Water Conservation Project, and the Yangtze River-Hanjiang River Diversion Project.

The Changjiang & Southeast Rivers Water Resources Bulletin (http://www.cjw.gov.cn/) shows that groundwater resources will account for only 1.9% of the total water supply in 2021. We used linear regression to predict future groundwater consumption (R^2 of 0.95) based on measured groundwater supply data from 2006-2021, and the threshold value is 1% in 2050 and 0.5% in 2050 under the optimal case.

Agricultural water use reduced by technology

Agriculture in the YZRC is improving irrigation efficiency by vigorously developing water-saving irrigation technologies. The improvement of water-saving irrigation technology is mainly reflected in the following aspects: promoting sprinklers, micro-irrigation, and drip irrigation instead of diffuse irrigation; using

pipeline impermeable water transmission instead of traditional earthen canals and rainwater harvesting to supplement irrigation, etc. The National Water Conservation Plan (http://ghjh.mwr.gov.cn/zdzt/qgszyzhgh/) predicts that the irrigation water use coefficient in 2030 will be greater than 0.6. Based on this, we assume a conservative case of 0.6 irrigation water use coefficient for farmland in the YZRC in 2030. The irrigation water use coefficient of the current water-saving irrigation methods in the YZRC is between 0.7 and 0.8. And according to the planning of the National Water Conservation Action Program[5], the basic penetration of water-saving irrigation can be fully achieved in 2050. Therefore, it is set to 0.7 in 2050, and the average irrigation water use per mu (mu, Chinese unit of land measurement that is commonly 666.7 m^2) of farmland is 370 m^3 and 350 m^3 in 2030 and 2050, respectively. In addition, a study by Niu et al. showed that future expansion of construction land in the YZRC would limit the area of arable land[6]. Therefore, this scenario is taken into account in the optimal case and the development of water-saving irrigation technologies for agriculture is increased after 2030, with the average irrigation water consumption per acre of farmland being 370 m^3 and 300 m^3 in 2030 and 2050, respectively.

The predicted equation for agricultural water use reduced by technical means is as follows:

$$Q_i = S * (W_a - W_i) \tag{2}$$

Where: $\boldsymbol{Q_i}$ denotes the agricultural water consumption reduced by technical means at the prediction time node in m^3; $\boldsymbol{S}$ denotes the irrigated area in mu; $\boldsymbol{W_a}$ denotes the multi-year average acreage irrigation water consumption from 2003 to 2018 in m^3/mu; $\boldsymbol{W_i}$ denotes the average acreage irrigation water consumption at the prediction time node in m^3/mu.

Industrial water use reduced by technology

The YZRC have now adopted a number of methods to reduce industrial water consumption. For example, recycling water resources, adopting efficient water-saving equipment and optimizing production processes, etc. The Industrial

Water Efficiency Improvement Action Plan (http://www.gov.cn/zhengce/zhengceku/) proposes that by 2025, the value-added water consumption of 10,000 yuan of industry will drop by 16%. Moreover, the Opinions on Implementing the Strictest Water Resources Management System[7] proposes that the value-added water consumption of industry will drop to less than 40 $m^3/10k$ (cubic meters per 10,000 Yuan) by 2030. Therefore, we conservatively give a value-added industrial water consumption of 40 $m^3/10k$ in the YZRC in 2030. It is presumed to be 30 $m^3/10k$ in 2050, and a conservative projection of 30 $m^3/10k$ in 2050. Under the optimal scenario, industrial water use efficiency in the YZRC would further improve to reach the level estimated in the Industrial Water Efficiency Improvement Action Plan - 94%, exceeding the 86% estimated by the Yangtze River Water Resources Commission. Therefore, the value-added industrial water use in the YZRC is set at 35 $m^3/10k$ in 2030 and 20 $m^3/10k$ in 2050.

Supplementary Note 4: Spatial matching of groundwater extraction and anthropogenic activities on the study unit

According to the current situation of groundwater use in China, groundwater sources are classified into discrete groundwater sources and centralized groundwater sources. Based on this, the groundwater supply situation in 27 provincial capitals and 4 municipalities directly under the central government in the Yangtze River catchment and Pearl River catchment was investigated. Most of the discrete groundwater sources are located in urban areas and support local domestic, agricultural, and industrial water use. The contribution of discrete groundwater sources to the overall water supply is relatively modest. Centralized groundwater sources are the main source of groundwater supply in each city. We investigated the official documents issued by each city and found that the straight-line distance between all sources and the urban area is less than the diameter of the study unit in this study (0.5°, about 55 km). This suggests that the grid cells of this study can meet the need for spatial matching between discharge areas and groundwater recharge areas (**Table S3**).

Supplementary Table:

Table S1 | Setting of carbon emission scenarios

Scenario	Year / Basins	2024 (%)	2026 (%)	2028 (%)	2030 (%)	2040 (%)	2050 (%)
CNS	RV	-27.0	-29.8	-32.7	-35.5	-60.0	-78.5
	GLB	-23.4	-30.0	-40.0	-50.0	-72.5	-88.7
	PRC	16.1	21.4	26.8	16.9	-22.3	-48.4
	YZRC	16.1	21.4	26.8	16.9	-22.3	-48.4
IPCC1.5B		15.6	25.0	34.4	43.8	56.3	68.8
IPCC1.5L		6.9	15.0	23.1	31.2	49.8	68.5
IPCC1.5W		7.0	15.2	23.4	31.6	50.6	69.6
IPCC1.5H		-2.5	2.5	7.5	12.6	34.6	56.6
IPCC2L		-2.5	2.5	7.6	12.6	31.5	50.5
IPCC2H		-3.0	0.0	3.0	6.0	24.0	42.0

Note: The CNS scenario uses 2005 as the base year for RV and GLB, while PRC and YZRC use 2018 as the base year. The six potential carbon emission scenarios established by the IPCC use 2010 as the base year.

Carbon emission categories: AGS--Agricultural soils; CHE--Agricultural waste burning; ENE--Energy industry; IND--Combustion in manufacturing industry; NEU--Non energy use of fuels; NFE--Production of non-ferrous metals; NMM--Production of non-metallic minerals; PRO--Fuel production/transmission; PRU_SOL--Production and use of other products; RCO—Residential; REF_TRF--Oil refineries; SWD_INC--Solid waste disposal; TNR--Non-road transport; TRO_noRES--Road transport.

Table S2 | Variation in all basins relative to AGWS at predicted time points

Basins	Scenarios \ Year	2024 (%)	2026 (%)	2028 (%)	2030 (%)	2040 (%)	2050 (%)
RV	CNS	5.04	5.19	5.51	5.57	5.45	9.26
	IPCC1.5B	3.92	4.67	5.77	6.09	5.26	7.08
	IPCC1.5L	3.18	3.75	4.37	5.39	5.41	7.13
	IPCC1.5W	3.18	3.91	4.47	5.51	5.48	7.54
	IPCC1.5H	1.96	2.61	3.27	3.66	5.80	5.38
	IPCC2L	1.97	2.64	3.24	3.68	5.40	5.43
	IPCC2H	1.81	2.24	2.71	3.12	4.62	6.22
GLB	CNS	0.65	1.21	2.25	3.32	6.50	11.02
	IPCC1.5B	-0.23	0.71	1.66	2.73	4.33	5.73
	IPCC1.5L	-0.81	-0.15	0.51	1.29	3.37	5.56
	IPCC1.5W	-0.88	-0.20	0.77	1.46	3.53	5.63
	IPCC1.5H	-1.61	-1.17	-0.85	-0.40	1.78	4.39
	IPCC2L	-1.70	-1.17	-0.68	-0.49	1.28	3.48
	IPCC2H	-1.67	-1.59	-1.08	-0.89	0.50	2.41
PRC	CNS	-0.01	-0.55	-1.31	-0.19	1.13	3.37
	IPCC1.5B	-2.28	-1.82	-1.06	-0.01	2.68	1.72
	IPCC1.5L	-2.68	-2.40	-1.91	-1.50	1.14	2.45
	IPCC1.5W	-2.88	-2.21	-1.93	-1.50	1.34	0.98
	IPCC1.5H	-2.85	-2.90	-2.73	-2.52	-1.16	2.80
	IPCC2L	-3.01	-2.94	-2.71	-2.43	-1.34	1.62
	IPCC2H	-3.03	-3.02	-2.87	-2.85	-1.89	-0.29
YZRC	CNS	0.43	-0.31	-1.17	-2.03	-6.94	-10.49
	IPCC1.5B	-5.13	-7.03	-8.94	-11.18	-14.46	-16.82
	IPCC1.5L	-2.13	-4.56	-6.56	-8.46	-12.57	-16.41
	IPCC1.5W	-1.83	-4.78	-6.84	-8.56	-12.71	-17.16
	IPCC1.5H	0.69	-0.96	-2.31	-3.74	-9.23	-14.53
	IPCC2L	0.82	-0.79	-2.32	-3.85	-8.55	-12.64
	IPCC2H	0.62	-0.12	-0.99	-1.84	-6.77	-10.33

Table S3 | Distance of centralized groundwater sources in the Yangtze and Pearl River basins from the urban areas of the cities they supply

Cities	Groundwater source areas	Distance of water source from water use area (km)
Shanghai	Groundwater extraction has been restricted and excluded from statistics	-
Chongqing	Surface water is the main source of water and excluded from statistics	-
Xining	Nanchuan, Beichuan and Sinagawa valleys	0
Xining	Chaiwuobao Six Water Source	45
Xining	Water source of Seven Water Source Plant and Ganhezi Water Source	44
Xining	Xishan Water Source	0
Lhasa	Dongjiao Water Treatment Plant Water Source	0
Lhasa	Beijiao Water Treatment Plant Water Source	0
Lhasa	Xijiao Water Treatment Plant Water Source	0
Lhasa	Yawangshan Water Treatment Plant Water Source	0
Chengdu	Surface water is the main source of water and excluded from statistics	-
Guiyang	Surface water is the main source of water and excluded from statistics	-
Kunming	Surface water is the main source of water and excluded from statistics	-
Nanning	Surface water is the main source of water and excluded from statistics	-
Guangzhou	Guangzhou Xijiang Diversion Water Source	28
Guangzhou	Nam Chau Water Treatment Works Water Source	28
Guangzhou	Dongjiang North Main Stream Water Source	0
Guangzhou	Water source on the south sand side of the Shawan waterway	0
Guangzhou	Tung Chung Water Treatment Works Water Source	0
Guangzhou	Shawan Water Treatment Plant Water Source	0
Guangzhou	Hong Xiuquan Reservoir	0
Guangzhou	Water source of the Shijiao section of the Liuxi River	0
Guangzhou	Water source of the Jiekou section of the Liuxi River	0
Guangzhou	Water source of Licheng section of Zengjiang River	0
Haikou	Surface water is the main source of water and excluded from statistics	-
Wuhan	Hankou Urban Water Source	0
Wuhan	WISCO Water Source	0
Wuhan	Xujiapeng Water Source	0
Wuhan	Baishazhou Water Source	0

Wuhan	Hanyang Water Source	0
Wuhan	Tianxingzhou Water Source	0
Wuhan	Huangpi Water Source	0
Changsha	Surface water is the main source of water and excluded from statistics	-
Nanjing	Surface water is the main source of water and excluded from statistics	-
Hefei	Surface water is the main source of water and excluded from statistics	-
Fuzhou	Surface water is the main source of water and excluded from statistics	-

Supplementary Figure:

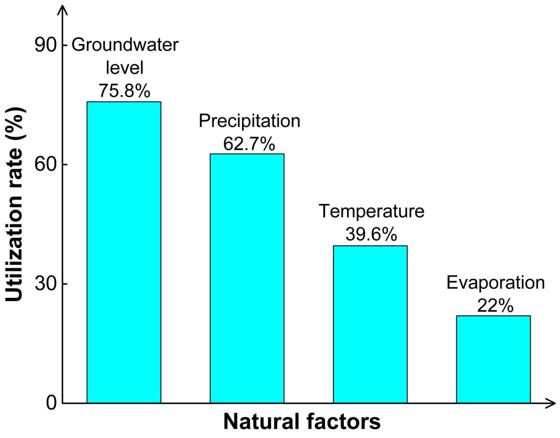

Supplementary Figure 1 | Utilization of various natural factors in groundwater prediction studies from 2000 to 2023.

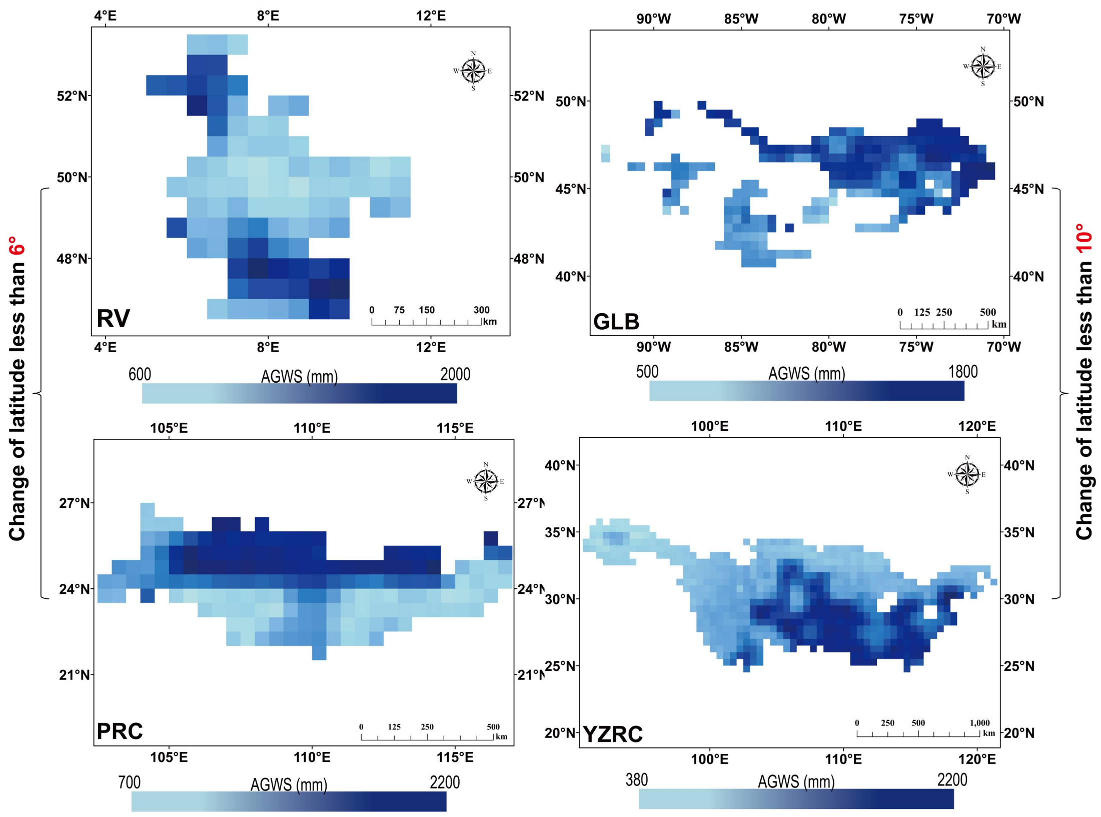

Supplementary Figure 2 | Spatial characteristics of groundwater storage for all basins. Spatial distribution of groundwater storage in order for RV, GLB, PRC and YZRC respectively (AGWS).

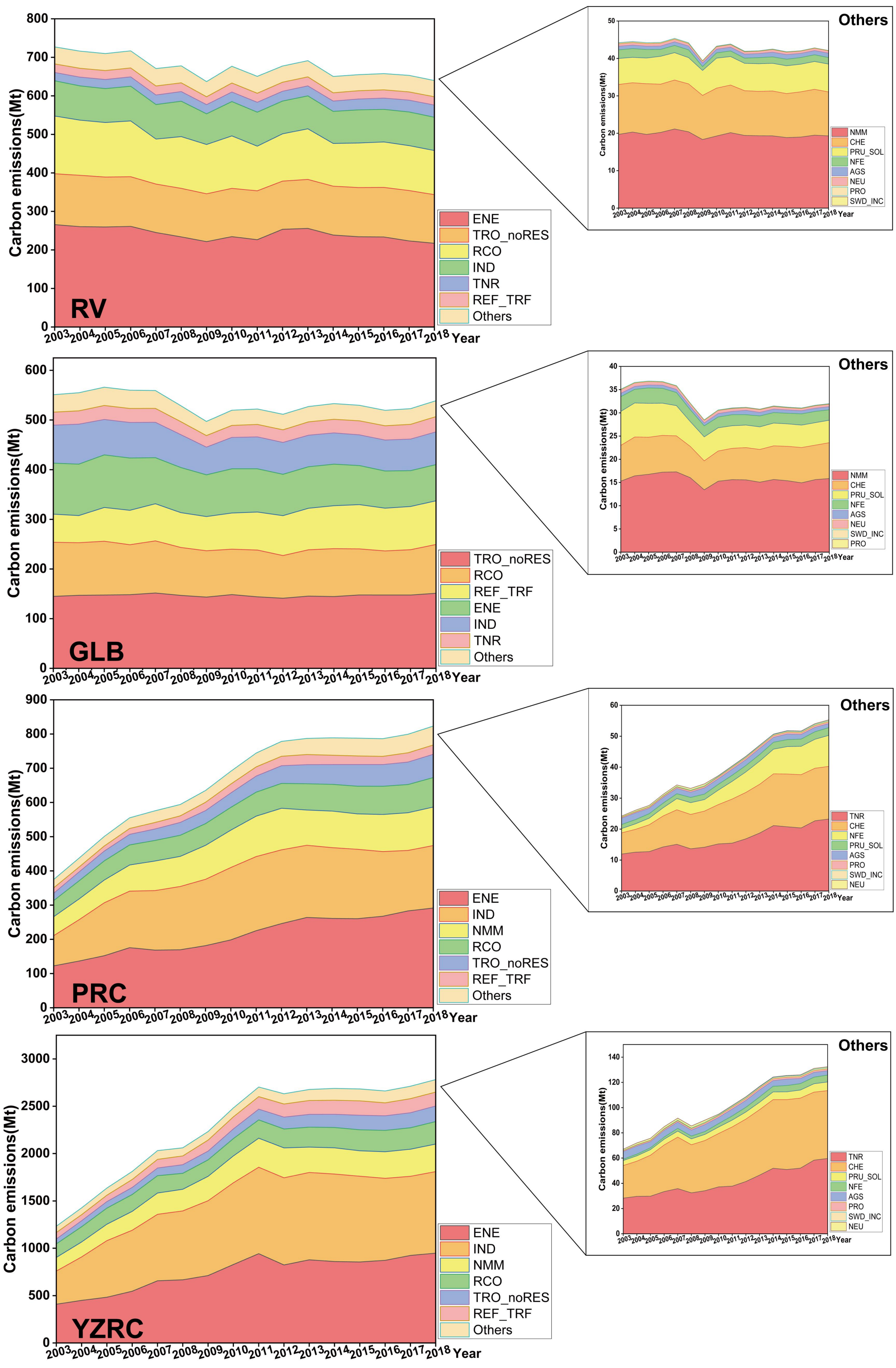

Supplementary Figure 3 | Carbon emissions variation in four basins.

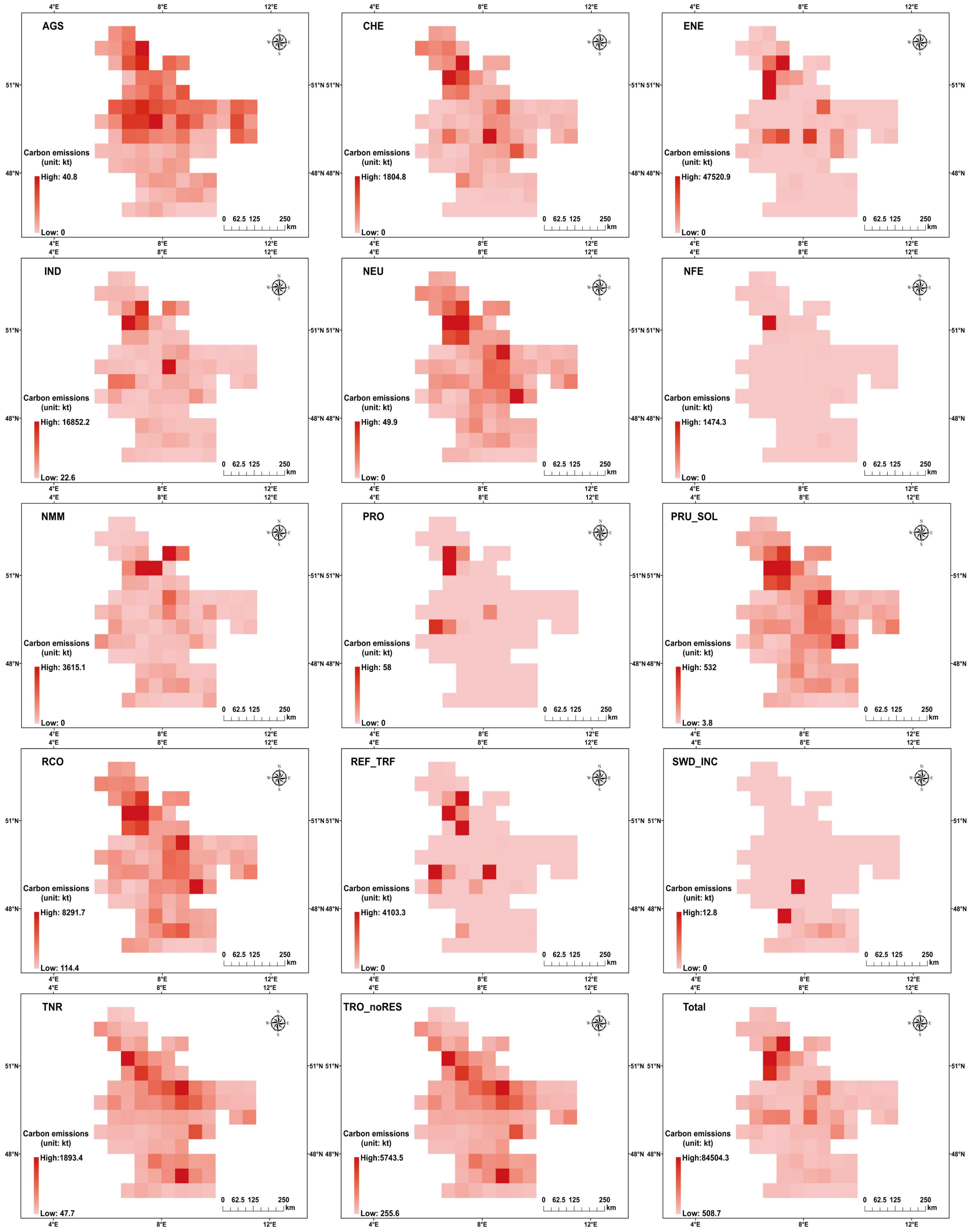

Supplementary Figure 4 | Spatial distribution of carbon emissions from 14 categories and total carbon emissions in RV. The graph shows the annual average carbon emissions for each grid from 2003 to 2018.

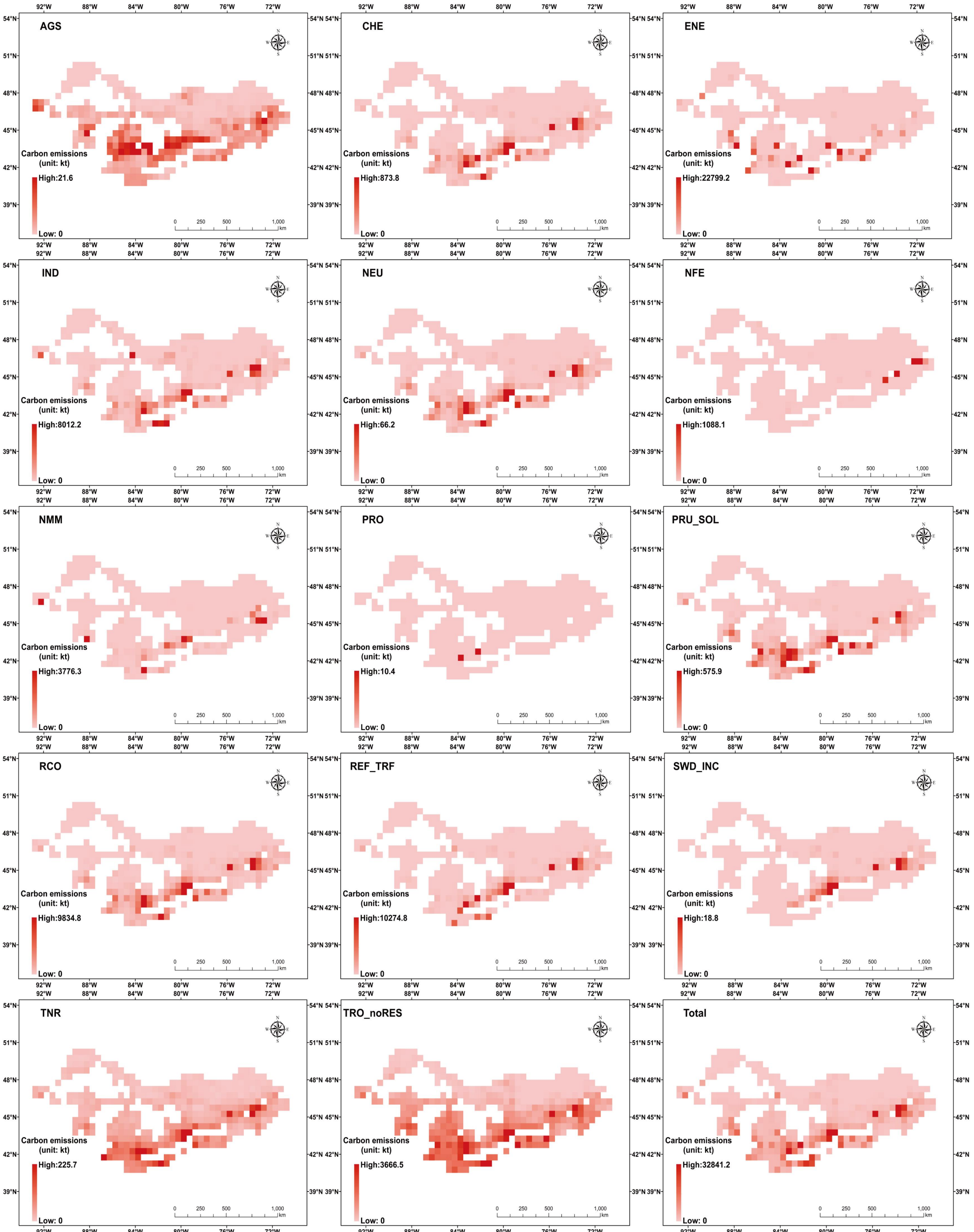

Supplementary Figure 5 | Spatial distribution of carbon emissions from 14 categories and total carbon emissions in GLB. The graph shows the annual average carbon emissions for each grid from 2003 to 2018.

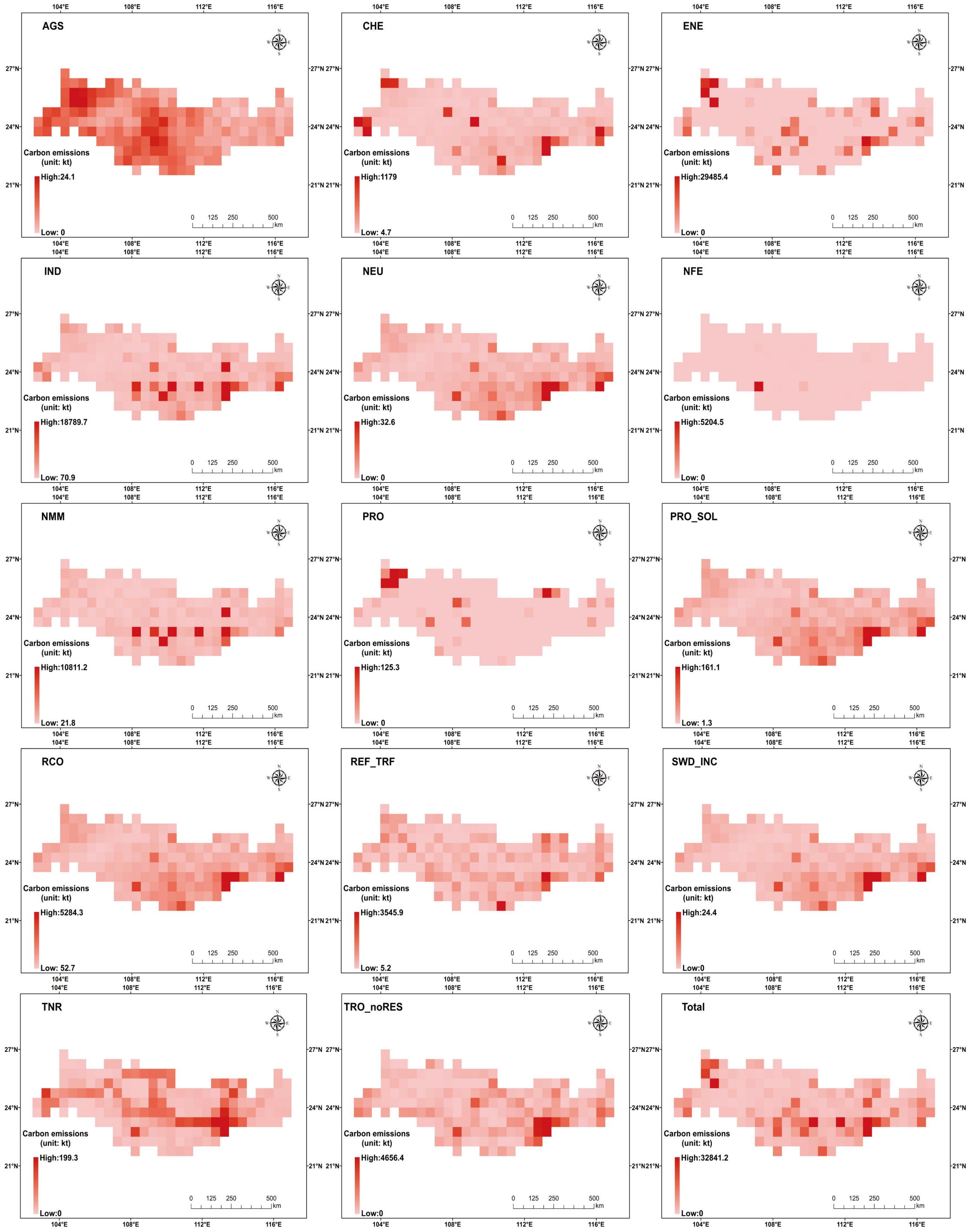

Supplementary Figure 6 | Spatial distribution of carbon emissions from 14 categories and total carbon emissions in PRC. The graph shows the annual average carbon emissions for each grid from 2003 to 2018.

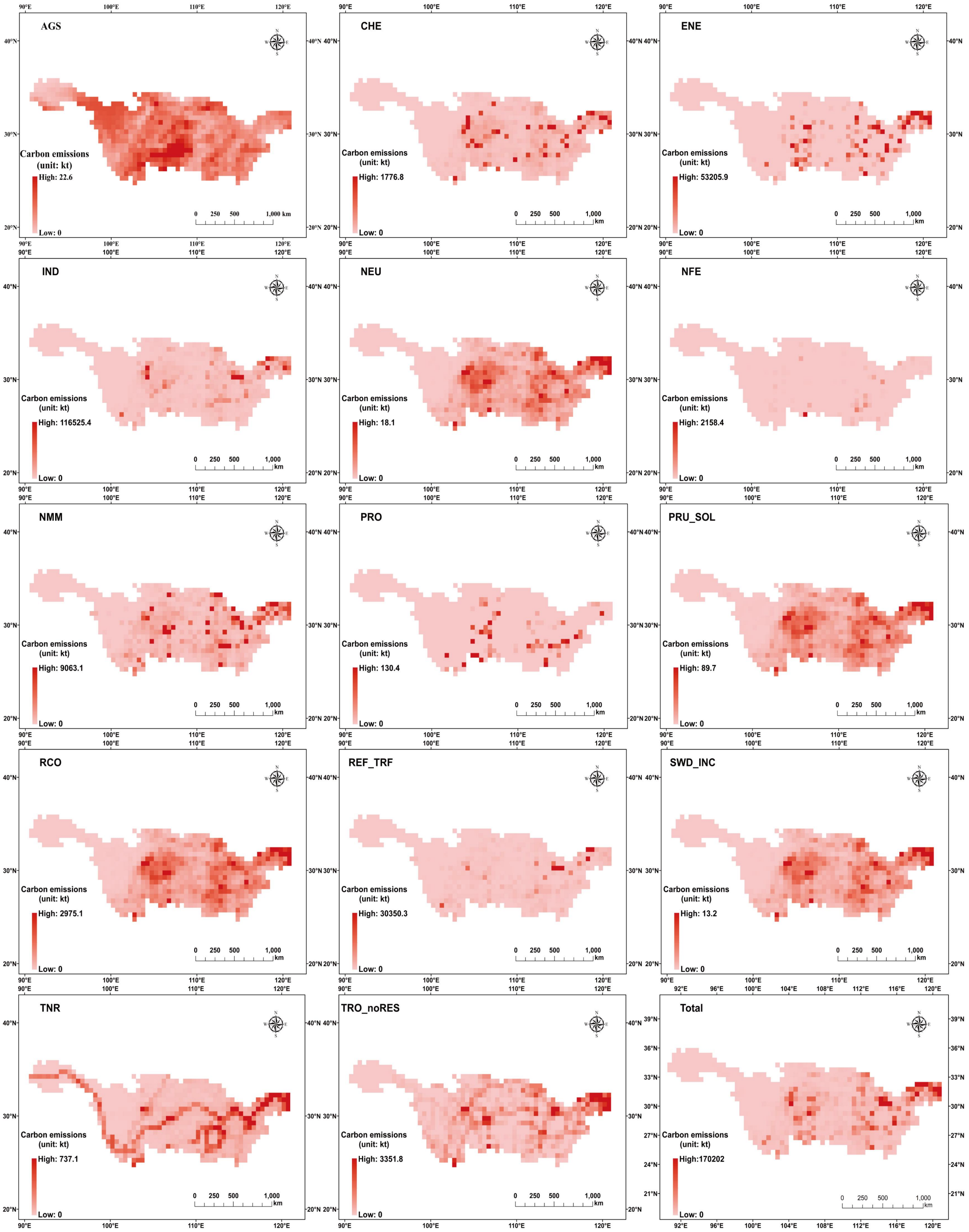

Supplementary Figure 7 | Spatial distribution of carbon emissions from 14 categories and total carbon emissions in YZRC. The graph shows the annual average carbon emissions for each grid from 2003 to 2018.

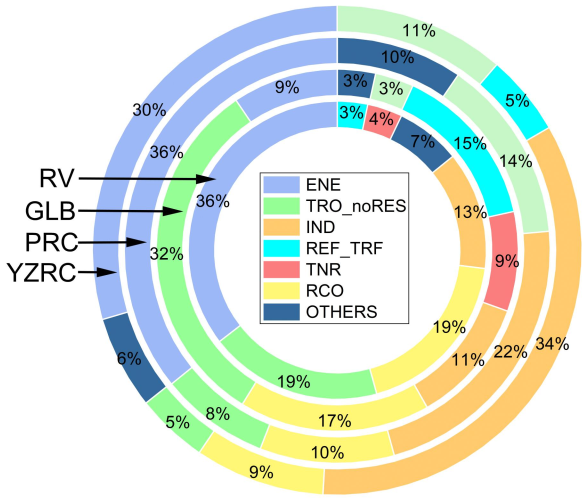

Supplementary Figure 8 | Proportion of carbon emissions by category in four basins.

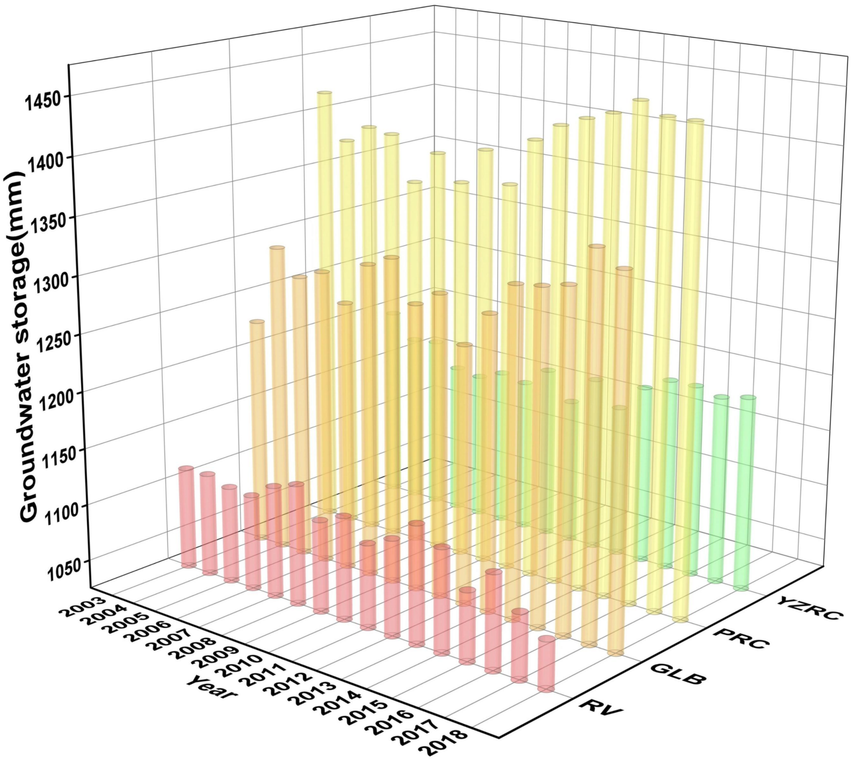

Supplementary Figure 9 | Groundwater storage variations from 2003 to 2018.

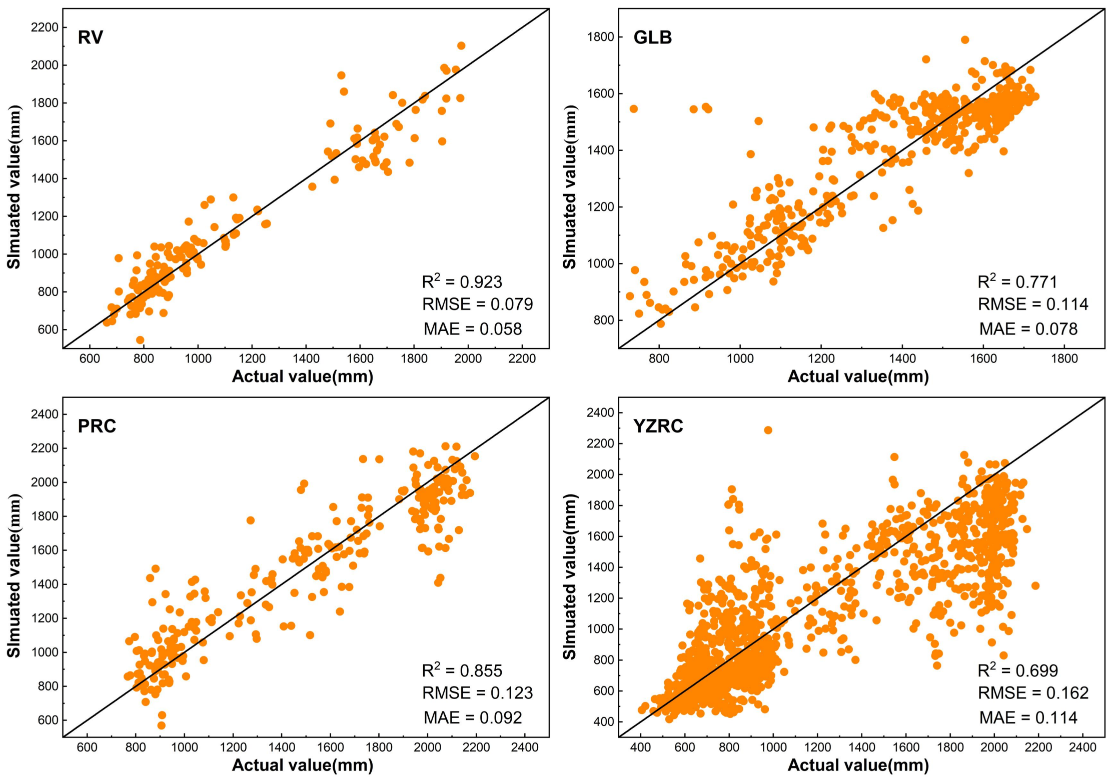

Supplementary Figure 10 | CNN model output for all basins test sets.

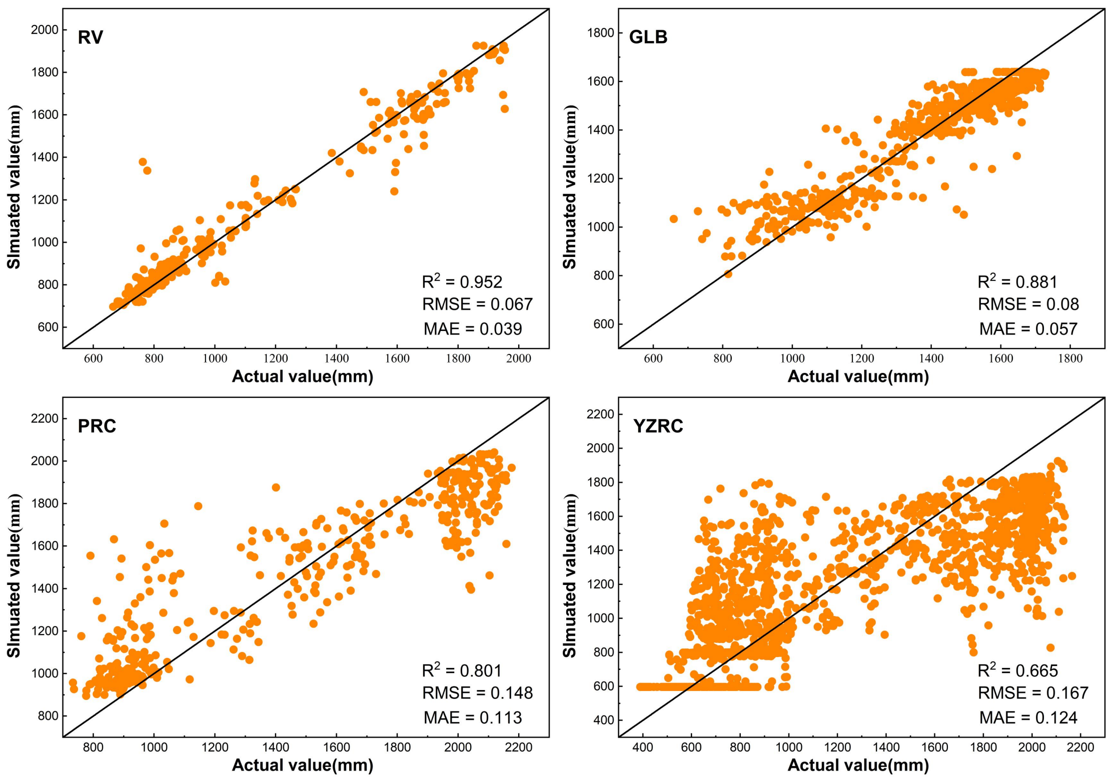

Supplementary Figure 11 | RF model output for all basins test sets.

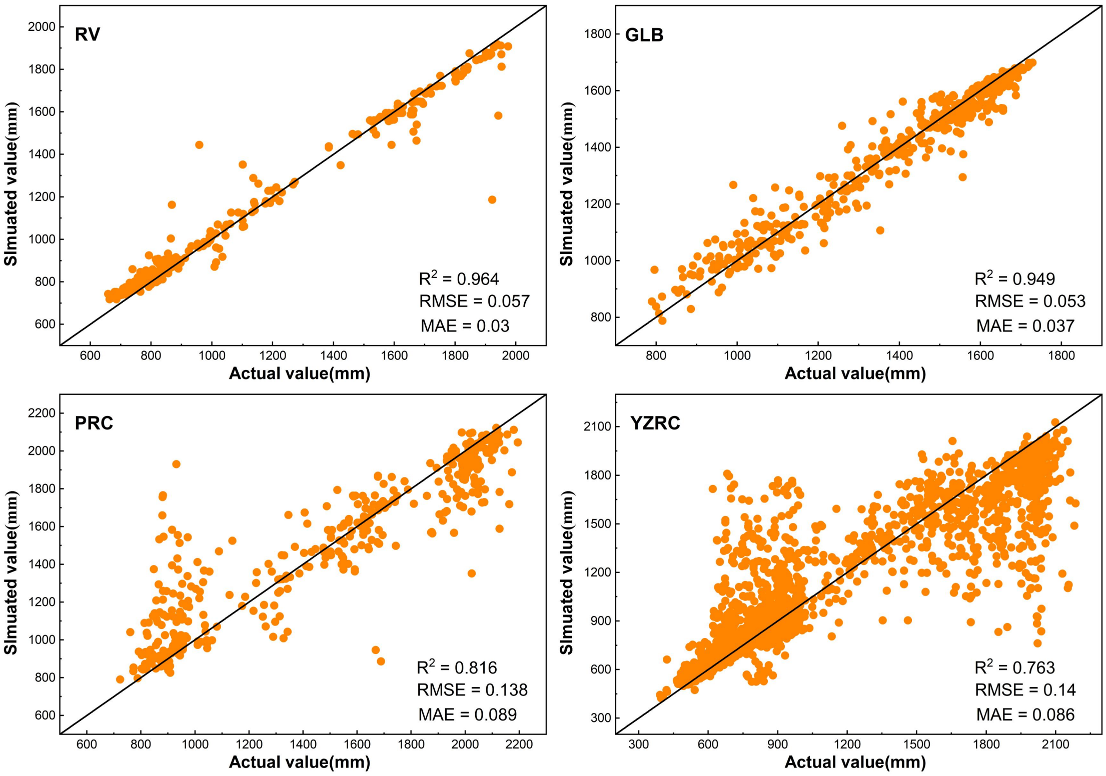

Supplementary Figure 12 | XGBoost model output for all basins test sets.

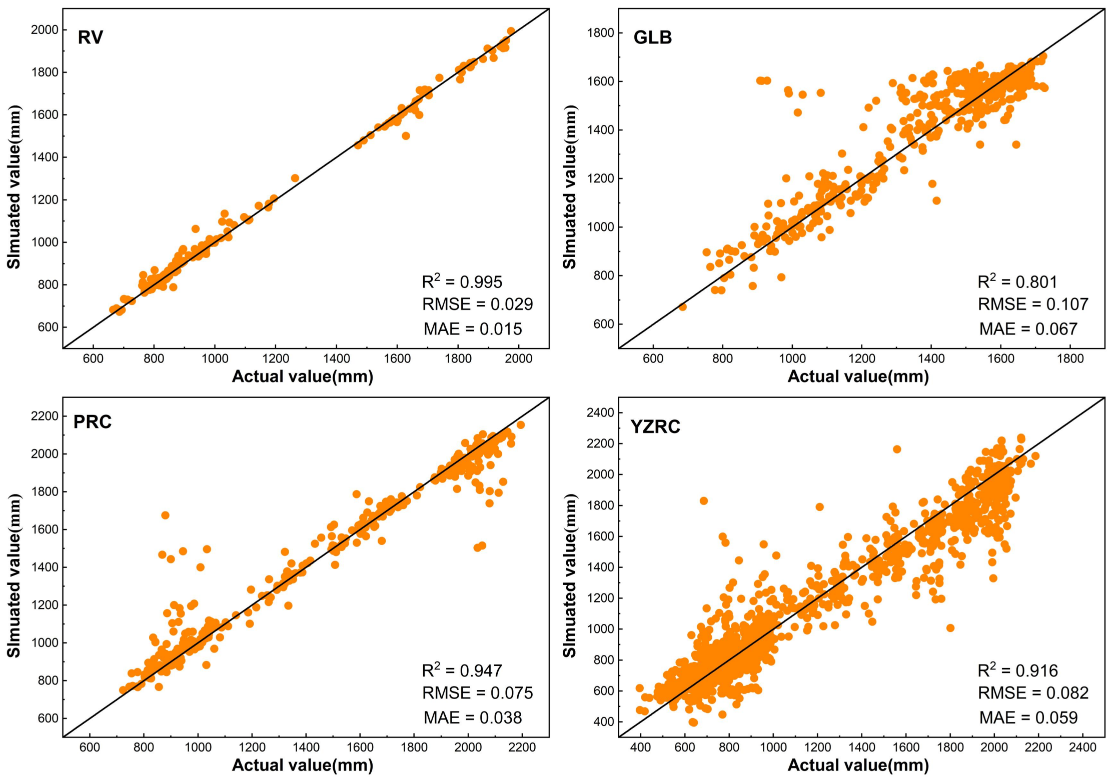

Supplementary Figure 13 | SVR model output for all basins test sets.

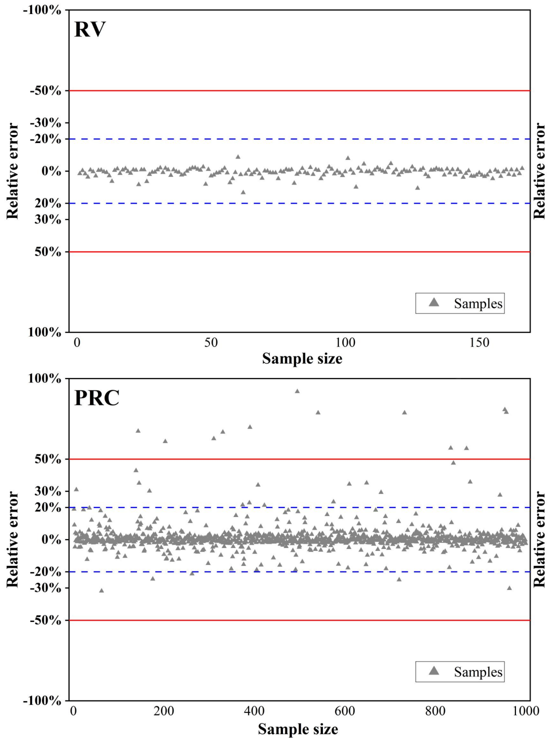

Supplementary Figure 14 | Relative errors in groundwater prediction models.

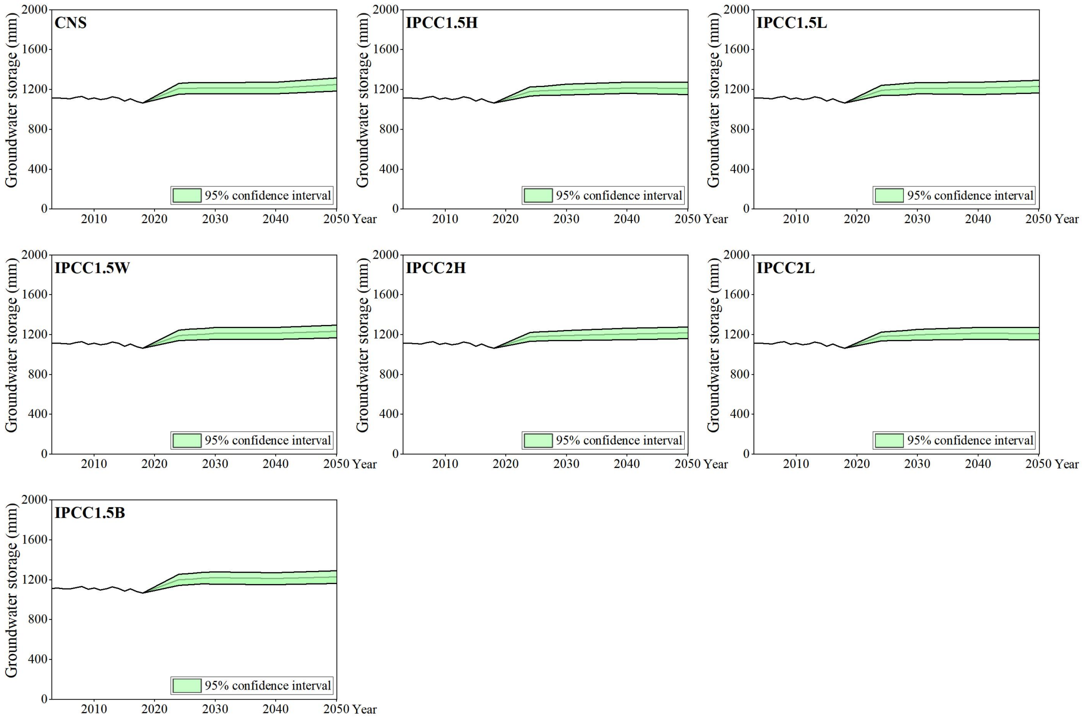

Supplementary Figure 15 | Predicted values of RV and results of uncertainty analysis.

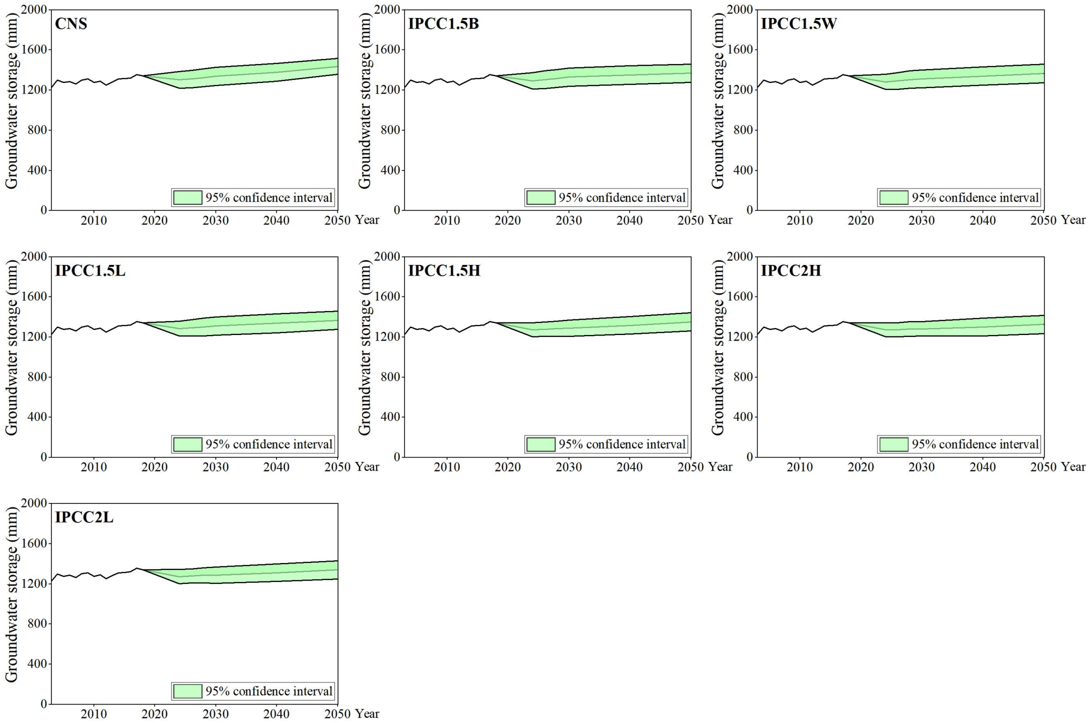

Supplementary Figure 16 | Predicted values of GLB and results of uncertainty analysis.

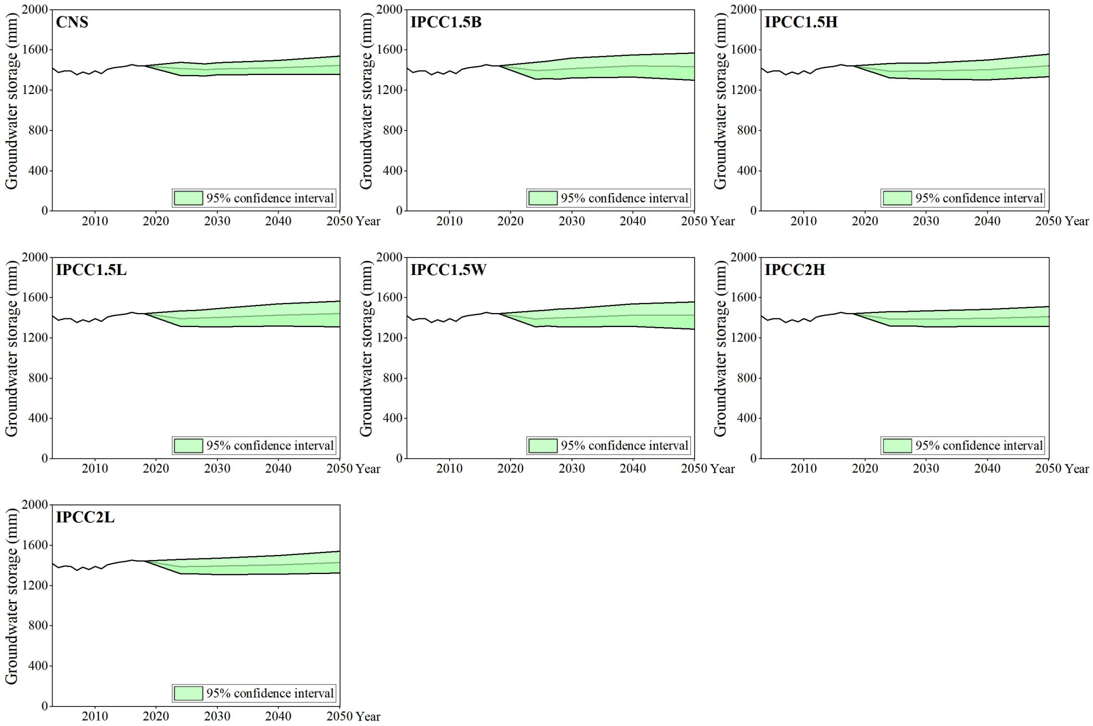

Supplementary Figure 17 | Predicted values of PRC and results of uncertainty analysis.

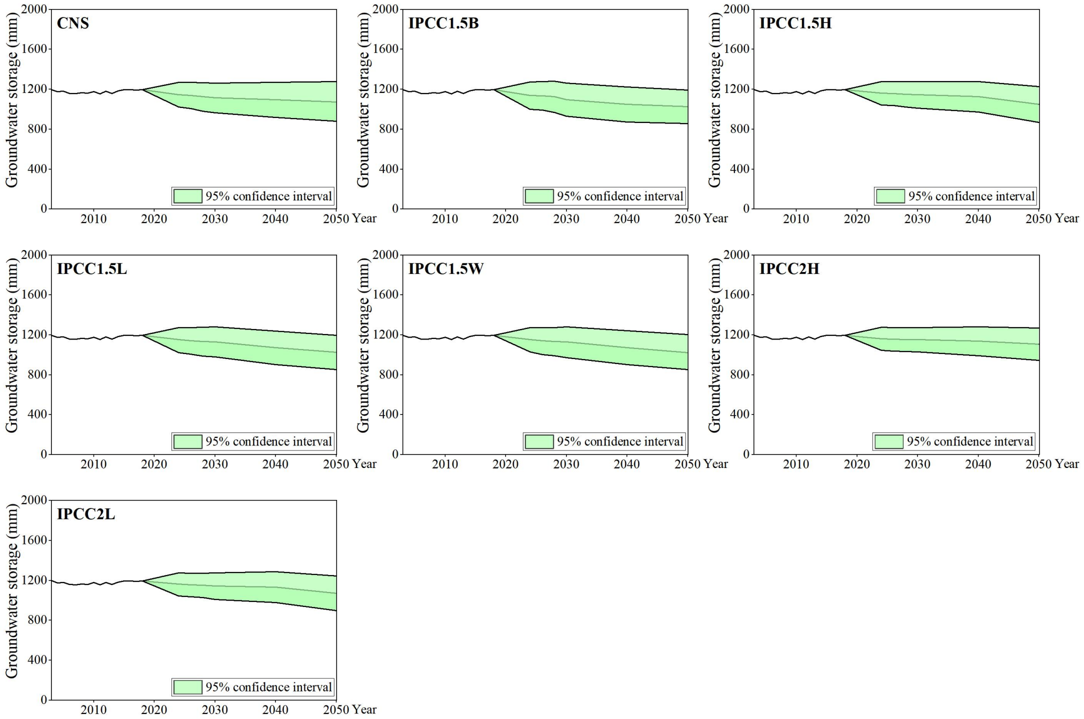

Supplementary Figure 18 | Predicted values of YZRC and results of uncertainty analysis.

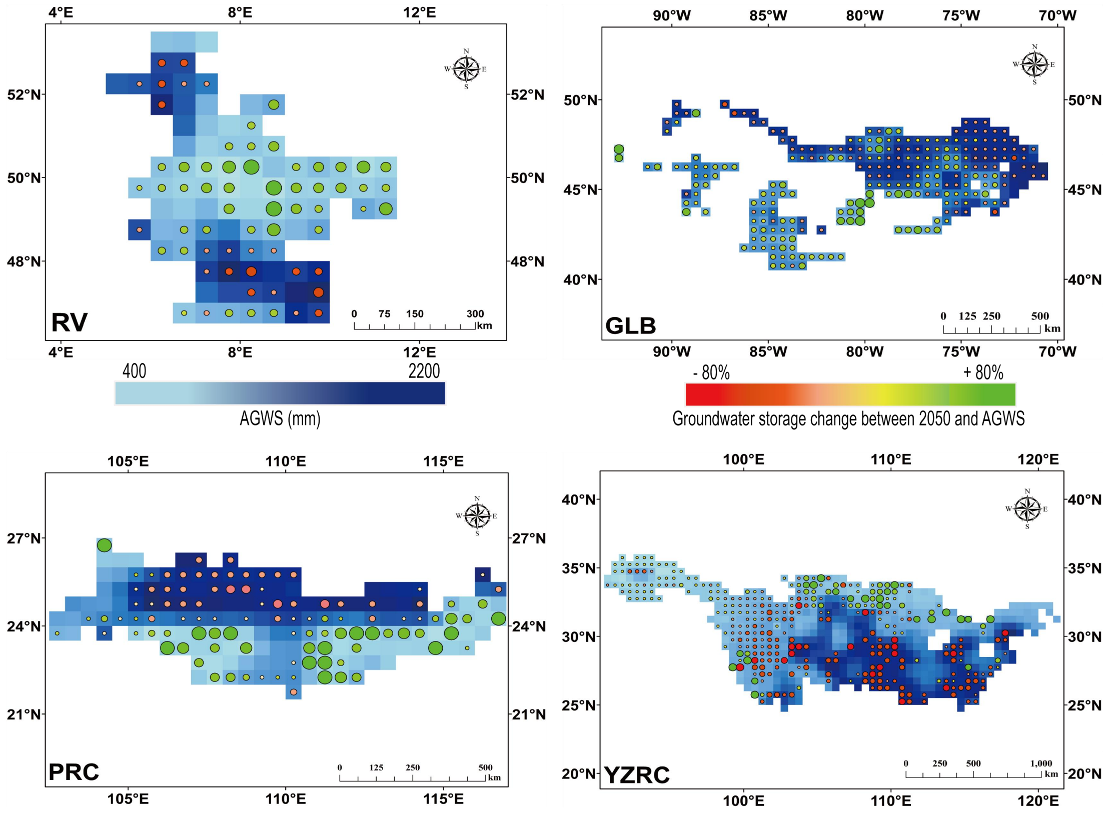

Supplementary Figure 19 | Groundwater storage variation in the IPCC1.5B. a, RV. **b,** GLB. **c,** PRC. **d,** YZRC. Change percentage in groundwater storage for each grid sample in 2050 relative to the multi-year average from 2003 to 2018. The size of the point indicates the magnitude of groundwater storage variation, the larger the point the greater the variation.

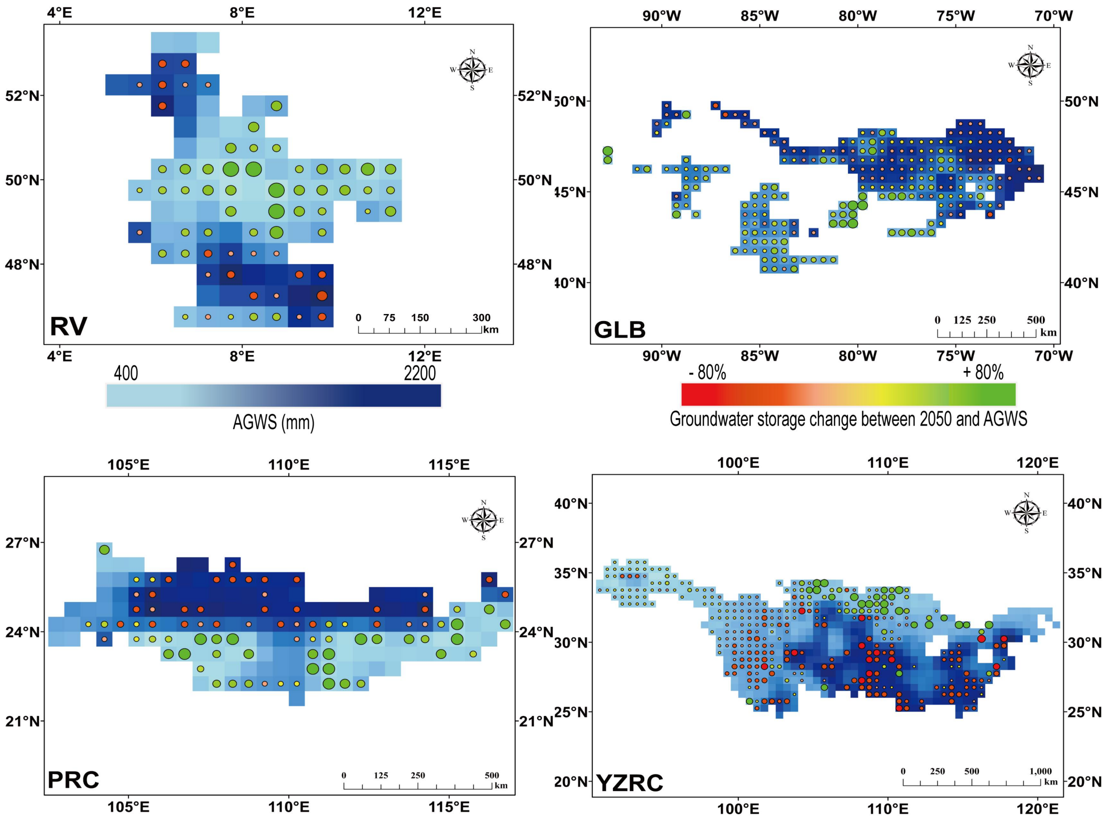

Supplementary Figure 20 | Groundwater storage variation in the IPCC1.5L.

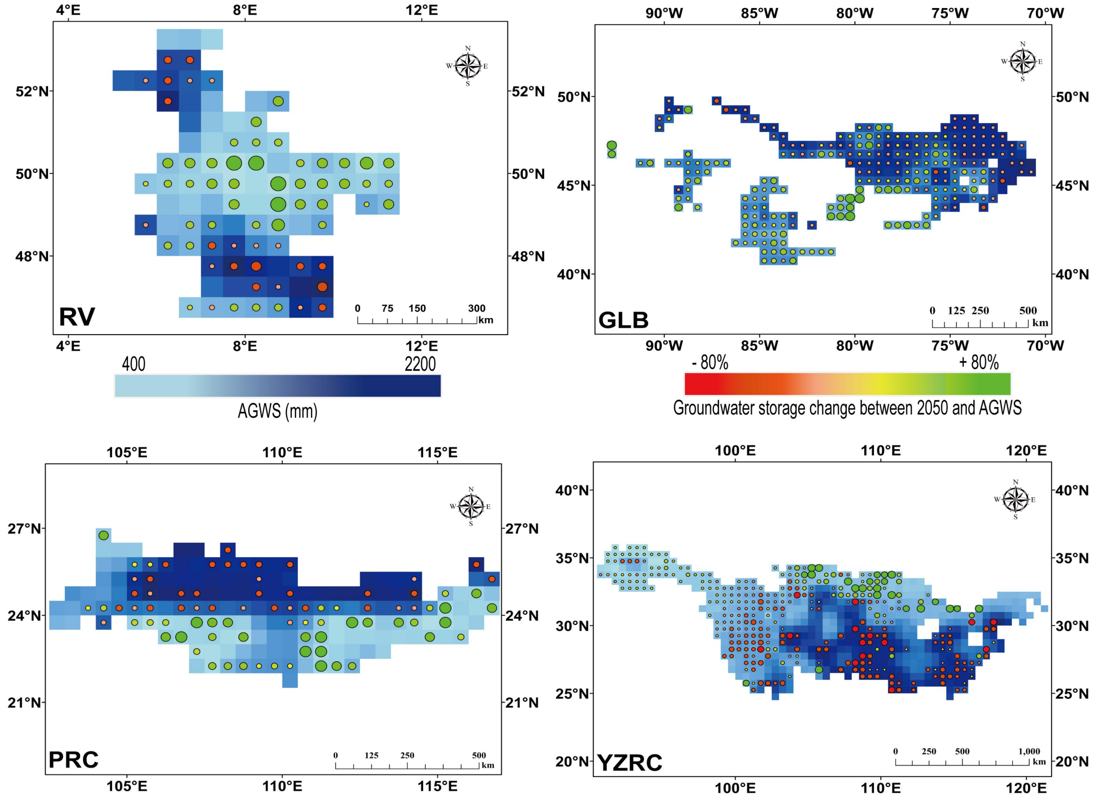

Supplementary Figure 21 | Groundwater storage variation in the IPCC1.5W.

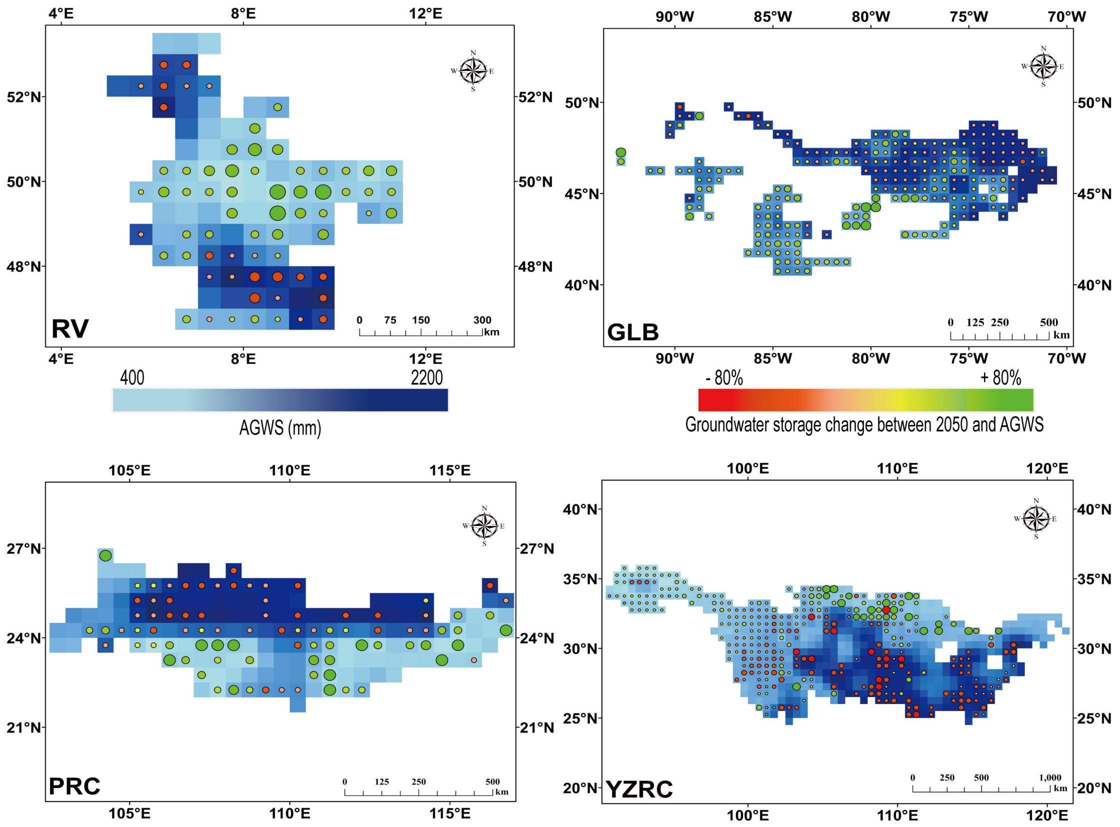

Supplementary Figure 22 | Groundwater storage variation in the IPCC1.5H.

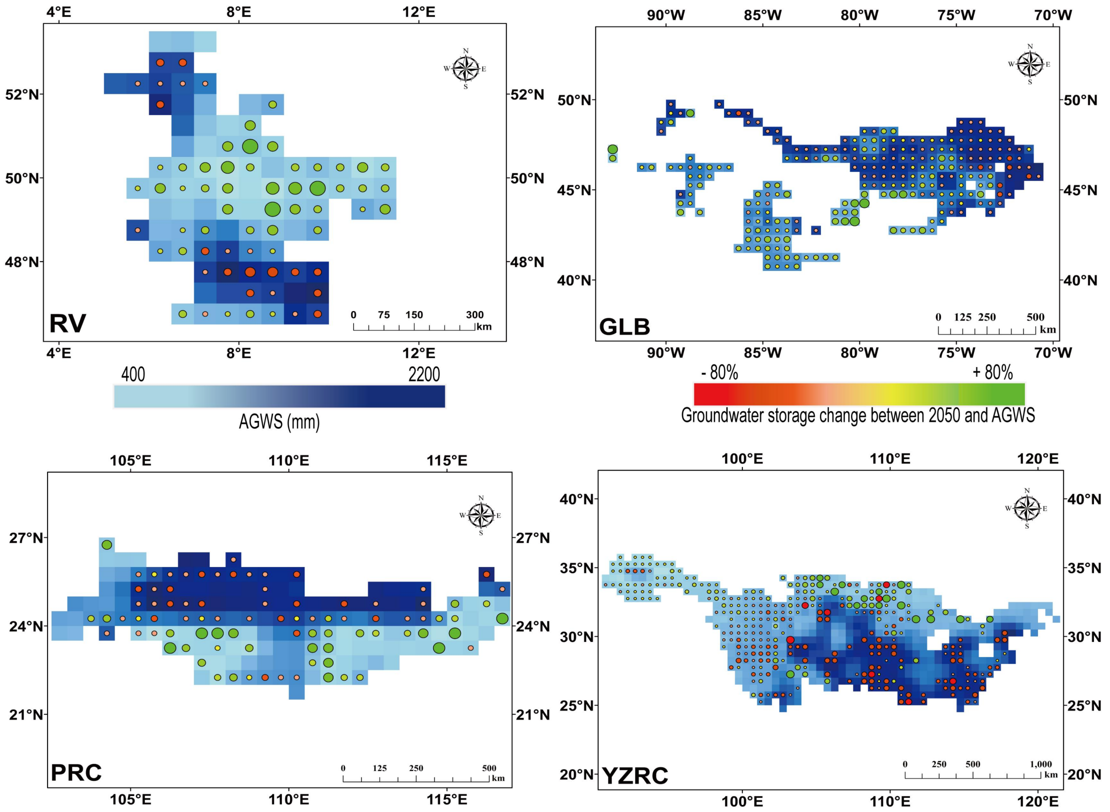

Supplementary Figure 23 | Groundwater storage variation in the IPCC2L.

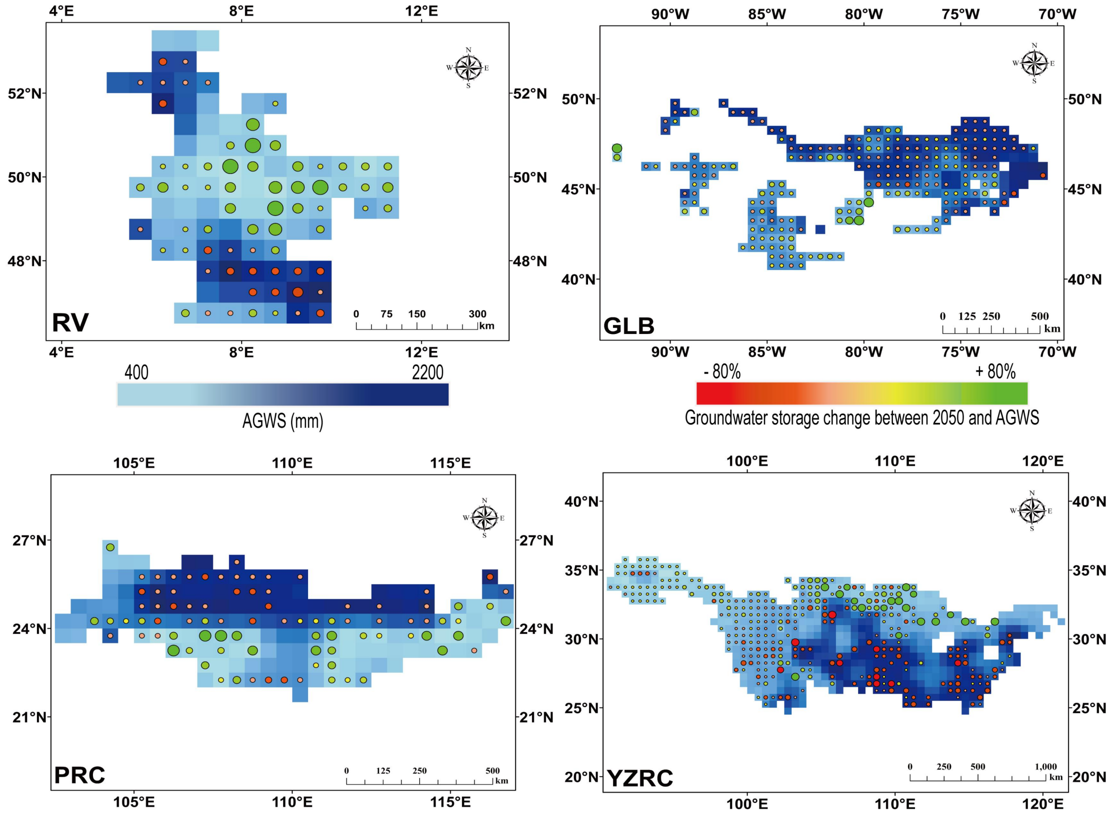

Supplementary Figure 24 | Groundwater storage variation in the IPCC2H.

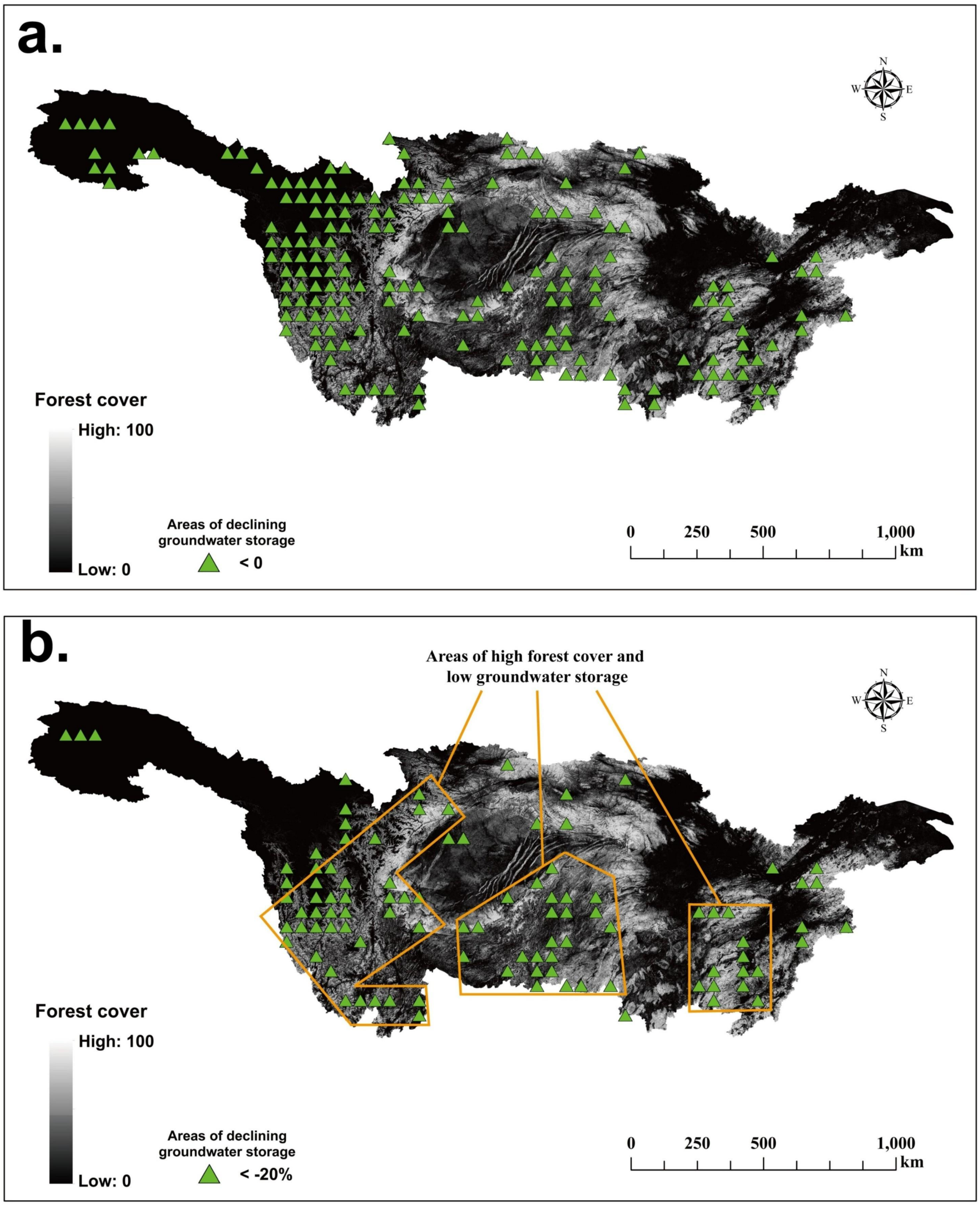

Supplementary Figure 25 | Forest cover and areas of declining groundwater storage. a. Diagram using 0% groundwater variation rate as the cut-off point. b. Diagram using 20% groundwater variation rate as the cut-off point. Forest cover sources: https://www.globalforestwatch.org.

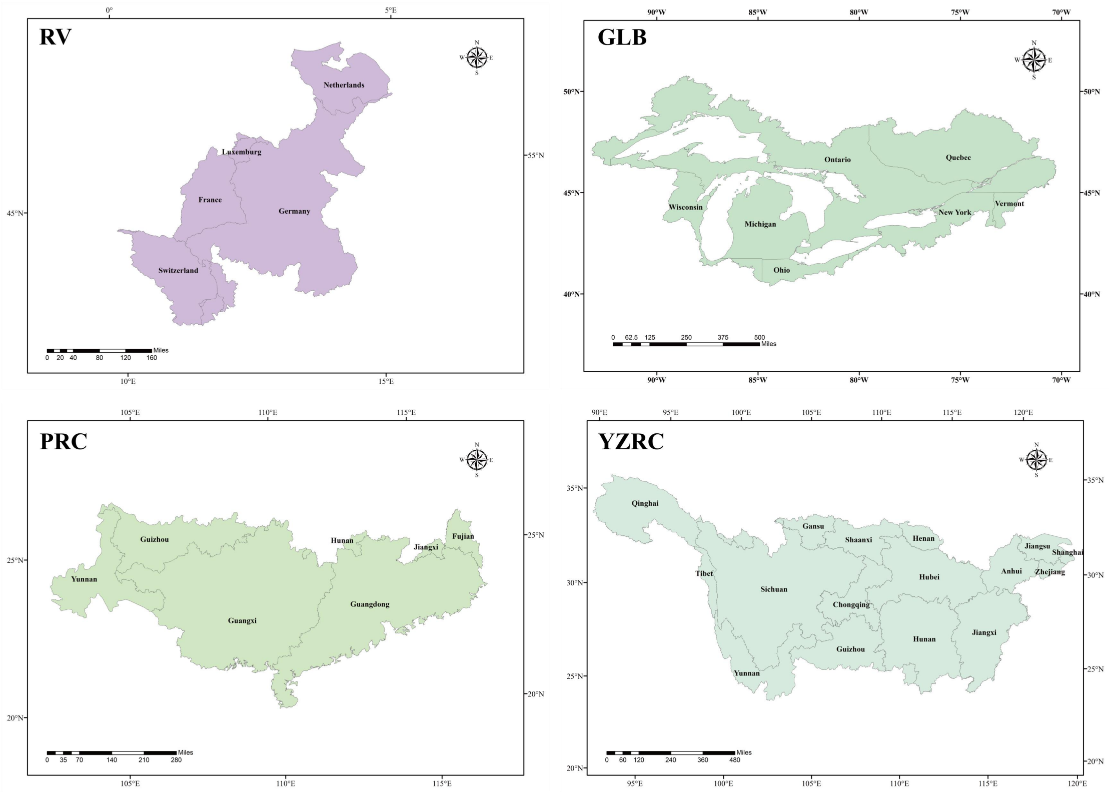

Supplementary Figure 26 | Basin Maps.

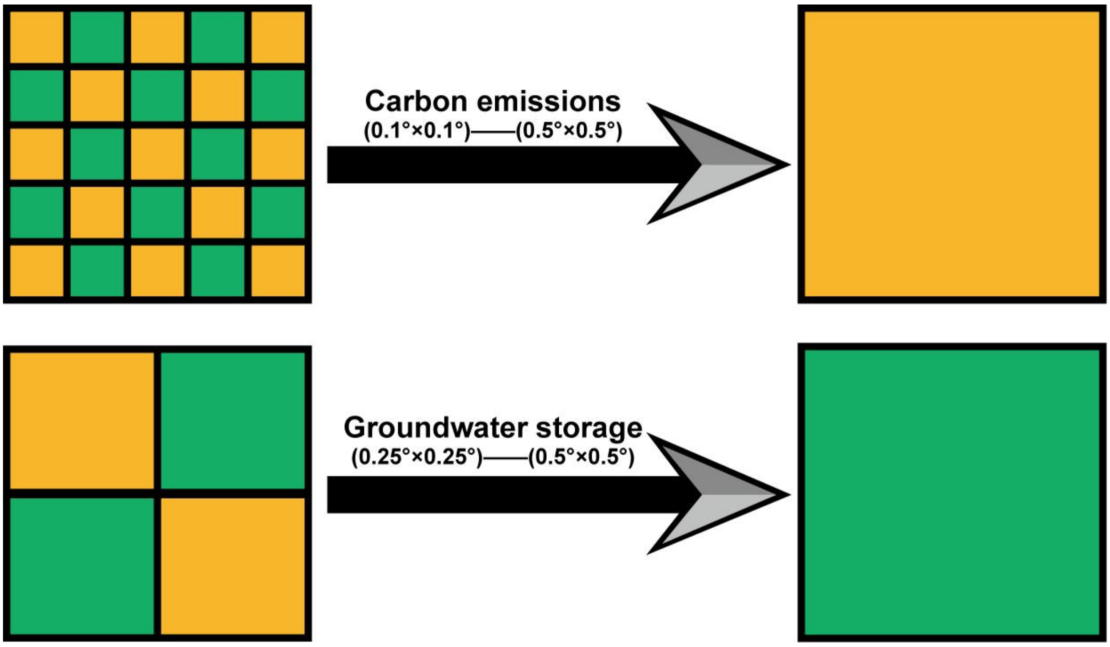

Supplementary Figure 27 | Grid sample data down-scaling diagram.

Supplementary References:

1 Wei, Y. *et al.* Roadmap for Achieving China's Carbon Peak and Carbon Neutrality Pathway. *J. Beijing Inst. Technol.* **24**, 13-26 (2022). (in Chinese)

2 Nalley, S. & LaRose, A. Annual energy outlook 2022 (AEO2022). *Energy Information Agency*, 23 (2022).

3 Capros, P., Tasios, N. & Marinakis, A. Very high penetration of renewable energy sources to the European electricity system in the context of model-based analysis of an energy roadmap towards a low carbon EU economy by 2050. *IEEE*, 1-8 (2012).

4 Masson D. V. *et al.* Global warming of 1.5 C. *An IPCC Special Report on the impacts of global warming of* **1**, 43-50 (2018).

5 Ministry of Water Resources of the People's Republic of China. National Water Conservation Action Program. 2019.

6 Niu, T., Xiong, L., Jie, C., et al. 2023. Land use simulation and multi-scenario prediction of the Yangtze River Basin based on PLUS model. Engineering Journal of Wuhan University (1671-8844). (in Chinese)

7 General Office of the State Council of the People's Republic of China. Opinions on Implementing the Strictest Water Resources Management System. 2012.

8 Moedinger J, Kobus H. Approach and Methods for the Assessment of Sustainable Groundwater Management in the Rhine–Neckar Region, Germany[J]. International journal of water resources development, 2005, 21(3):p.437-451.

9 Calatrava J, Martínez-Granados, David. Water buybacks to recover depleted aquifers in south-east Spain[J]. International Journal of Water Resources Development, 2018:1-22.

10 Rouillard J, Babbitt C, Pulido-Velazquez M, et al. Transitioning out of Open Access: A Closer Look at Institutions for Management of Groundwater Rights in France, California, and Spain[J]. Water Resources Research, 2021(4).

11 Mitter H, Schmid E. Informing groundwater policies in semi-arid agricultural production regions under stochastic climate scenario impacts[J]. Ecological Economics, 2021, 180:106908.

12 Jrgensen L F, Villholth K G, Refsgaard J C. Groundwater management and protection in Denmark: a review of pre-conditions, advances and challenges[J]. International Journal of Water Resources Development, 2016, 33:1-22.
